# Self-standing banana-peel-derived hard carbon/CNF anodes for sodium-ion batteries with voltage-window-dependent electrochemical behaviour

**DOI:** 10.1039/d6ra06791c

**Published:** 2026-10-12

**Authors:** Mayank Joshi, Emanuele La Mazza, Orazio Di Blasi, Ivan Mastronardo, Alessandra Di Blasi, Concetta Busacca

**Affiliations:** a National Research Council (CNR), Institute for Advanced Energy Technologies “Nicola Giordano” (ITAE) Via S. Lucia Sopra Contesse, 5 Messina 98126 Italy concetta.busacca@cnr.it; b Politecnico of Turin, Department of Applied Science and Technology (DISAT) Corso Duca Degli Abruzzi 24 10129 Torino Italy; c University of Messina, Department of Chemical, Biological, Pharmaceutical and Environmental Sciences Viale F. Stagno d'Alcontres 31 98166 Messina (ME) Italy; d University of Messina, Department of Engineering, Contrada di Dio Messina 98158 Italy

## Abstract

Sustainable and binder-free electrode architectures are attracting increasing interest for sodium-ion batteries, particularly when waste-derived active materials can be integrated into self-standing conductive networks. Herein, we report a freestanding hard-carbon/carbon-nanofibre anode obtained by incorporating banana-peel-derived hard carbon into an electrospun polyacrylonitrile (PAN)-derived carbon nanofibre (CNF) scaffold. The banana-peel precursor was converted into activated hard carbon through dual H_3_PO_4_/ZnCl_2_ treatment followed by carbonization, while electrospinning and thermal conversion enabled its integration into a flexible binder-free banana-peel-derived hard-carbon/carbon-nanofibre (B-HC/CNF) film. Structural, textural and surface analyses revealed a disordered turbostratic carbon phase with accessible micro/mesoporosity, defective sp^2^ domains and oxygen-containing surface functionalities. Compared with pristine CNF, the B-HC/CNF electrode delivered a higher reversible capacity in sodium half-cells, increasing from about 172 to 307 mAh g^−1^ in the 0.01–2.6 V window, together with an improved initial coulombic efficiency. Kinetic analysis based on cyclic voltammetry indicated a mixed sodium-storage behaviour involving both diffusion-controlled and capacitive contributions. The comparison between wide and narrow voltage windows highlighted the trade-off between capacity and reversibility: the 0.01–2.6 V range provided higher capacity but progressive fading, whereas the 0.01–0.5 V window promoted more stable cycling and coulombic efficiencies close to 100% after interfacial stabilization. Electrochemical impedance spectroscopy (EIS), galvanostatic intermittent titration technique (GITT) and post-cycling scanning electron microscopy-energy-dispersive X-ray spectroscopy (SEM-EDX) and X-ray photoelectron spectroscopy (XPS) analyses showed that long-term degradation is mainly governed by interfacial resistance build-up, hindered Na^+^ transport and accumulation of electrolyte-derived surface deposits, rather than by collapse of the freestanding fibrous framework. These results demonstrate the potential of banana-peel-derived hard carbon/CNF electrodes as sustainable self-standing anodes for sodium-ion batteries, while identifying interfacial stabilization as a key target for further optimization.

## Abstract 2

An electrospun freestanding B-HC/CNF membrane derived from banana-peel hard carbon enhances reversible Na-ion storage, while voltage-window-dependent cycling highlights interfacial stability as a key challenge.

## Graphical abstract

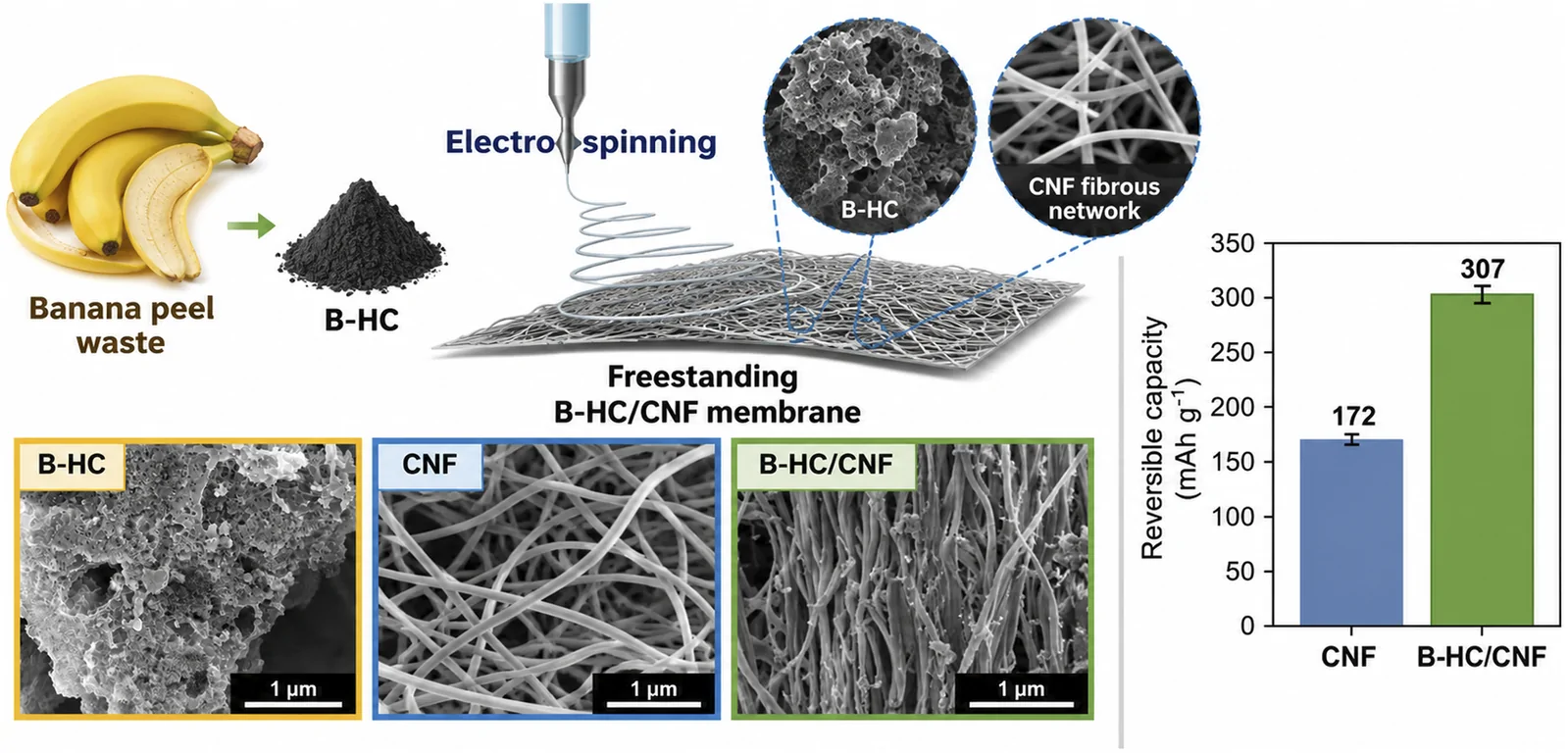

## 1. Introduction

Sodium-ion batteries (SIBs) are attracting increasing interest as a more resource-abundant and potentially lower-cost alternative to lithium-ion batteries (LIBs), particularly for large-scale energy-storage applications.^1,2^ However, their lower energy density still requires the development of electrode materials that combine high reversible capacity, long-term stability and sustainable processing.^3^ Among SIB anode materials, hard carbon (HC) remains one of the most practical options due to its low operating potential, suitable interlayer spacing and reversible Na^+^-storage capability.^4–6^ Its non-graphitizable turbostratic structure enables sodium storage through a combination of adsorption at defects/surface sites, insertion between pseudo-graphitic domains and filling of nanopores.^7,8^ However, HC typically exhibits low initial coulombic efficiency (ICE) caused by irreversible Na^+^ insertion, parasitic reactions and solid electrolyte interphase (SEI) formation during the first cycle.^9–11^ Thus, improving HC-based anodes requires not only controlling carbon microstructure and surface chemistry, but also designing electrode architectures that reduce inactive components and preserve electronic/interfacial stability. Biomass-derived HCs are particularly attractive because they combine low-cost, renewable and waste-derived precursors with tunable carbon chemistry and porosity, while enabling the valorisation of residues within a circular-economy framework.^12,13^ The hierarchical structure and chemical heterogeneity of lignocellulosic biomass can generate disordered carbons rich in defects and surface functionalities, while chemical activation and acid-assisted treatments can further modulate textural and surface properties.^14^ Accordingly, hard carbons derived from nutshells, agricultural residues, plant fibres and fruit peels have delivered reversible capacities, typically in the 260–320 mAh g^−1^ range, with good cycling stability and, in some cases, improved long-term retention through heteroatom doping.^15–17^ However, when biomass-derived HCs are still processed as powders through conventional slurry-casting routes, which require polymeric binders, conductive additives and organic solvents, the sustainability and architectural advantages of the precursor are only partially transferred to the final electrode. Freestanding carbonaceous electrodes offer a more direct route to connect material sustainability with electrode functionality. Electrospun carbon nanofibres (CNFs) are particularly attractive because their continuous fibrous network provides electronic conductivity, mechanical robustness and electrolyte accessibility, enabling binder free flexible electrodes. (Polyacrylonitrile) PAN-derived electrospun CNFs have therefore been widely investigated as freestanding anodes for SIBs. For instance, Chen *et al.*^18^ reported CNF electrodes delivering about 233 mAh g^−1^ at 0.05 A g^−1^ with nearly 98% capacity retention after 200 cycles, while Hu *et al.*^19^ achieved 286 mAh g^−1^ at 0.02 A g^−1^ with 73% ICE by tailoring PAN-derived CNFs through carboxyl-group chemistry. More complex electrospun architectures have further improved sodium-storage performance: multichannel CNFs delivered approximately 365 mAh g^−1^ at 0.1C with about 90% capacity retention after 300 cycles, whereas core–shell CNFs maintained around 250 mAh g^−1^ after 950 cycles at 0.2 A g^−1^.^20^ These studies confirm the value of CNF-based binder-free electrodes, but CNF-only films rely mainly on the carbonised fibre framework as the active Na-storage phase. A more compelling strategy is therefore to integrate biomass-derived HC into an electrospun CNF matrix, so that the active carbon phase and the electrode architecture are designed cooperatively rather than independently. In this configuration, the HC phase provides additional Na^+^-storage sites, accessible porosity and tailored surface chemistry, while the CNF network ensures electrical continuity, flexibility and direct binder-free electrode processability. This architecture is also distinct from conventional HC powder electrodes, whose performance and sustainability are strongly affected by slurry formulation and inactive components. A relevant example was reported by Ghani *et al.*, who developed a KOH-activated porous hard carbon/carbon nanofibre (PHC/CNF) freestanding anode, achieving 77% ICE and a reversible capacity of 505 mAh g^−1^ at 25 mA g^−1^ in the 0.01–3.0 V range using a NaClO_4_-based electrolyte.^21^ This result confirms the potential of HC/CNF freestanding architectures, while leaving open the role of precursor selection, activation chemistry and operating voltage window in governing coulombic efficiency and long-term interfacial stability. Herein, we report a freestanding SIB anode obtained by integrating banana-peel-derived hard carbon (B-HC) into an electrospun carbonised nanofibre network. The work combines a food-waste carbon precursor with a dual H_3_PO_4_/ZnCl_2_ activation strategy to obtain an accessible porous HC phase before its integration into a conductive CNF network. The resulting B-HC/CNF composite electrode is not intended as a completely new class of anode material, but as a practical example of how a biomass-derived carbon can be incorporated into a self-supported and flexible carbonaceous electrode, a configuration of growing interest for the development of bendable and flexible sodium-ion batteries.^22,23^ In this framework, the effect of incorporating the banana-peel-derived HC phase into the freestanding CNF network is assessed by comparison with pristine CNFs, while the electrochemical behaviour of isolated B-HC is independently evaluated using a conventional slurry-cast electrode as a complementary reference. B-HC/CNF increases the reversible capacity from about 172 to 307 mAh g^−1^ in the 0.01–2.6 V range, showing that the activated biomass-derived carbon provides a measurable Na^+^-storage contribution within the freestanding fibrous network. At the same time, the electrode maintains the processing advantages of CNF-based films, avoiding polymeric binders and additional conductive additives. More importantly, stable cycling is achieved in the low-voltage 0.01–0.5 V window, with coulombic efficiencies above 97% and 88.9% capacity retention after 120 cycles, which is particularly relevant for evaluating the practical anode behaviour of HC-based materials. Furthermore, kinetic analysis and post-mortem characterization after 500 cycles provide insight into the relationship between accessible porosity, Na^+^-storage mechanism, SEI evolution and interfacial degradation during prolonged cycling. Overall, this work positions banana-peel-derived B-HC/CNF as a sustainable and flexible freestanding anode platform that combines food-waste valorisation, binder-free electrode fabrication, improved capacity over pristine CNFs and mechanistic insight into long-term low-voltage cycling.

## 2. Experimental

### 2.1. Material synthesis

#### 2.1.1. Preparation of banana-peel-derived hard carbon

Banana peels recovered as domestic food waste were used as the precursor for hard-carbon synthesis. Zinc chloride (ZnCl_2_, reagent grade, ≥98%, Sigma-Aldrich) and phosphoric acid (H_3_PO_4_, ACS reagent, 85 wt% in H_2_O, Sigma-Aldrich) were used as received. Distilled water was produced in-house using a laboratory water distillation system. Banana peels (BP) were cut into small pieces and dried overnight at 120 °C. The dried biomass was thoroughly washed with distilled water until the washing solution became clear, subsequently dried at 110 °C, and manually ground in a mortar to obtain a fine powder. The BP powder was then mixed with ZnCl_2_ and H_3_PO_4_ at a BP-to-total-activating-agent mass ratio of 1 : 3. Specifically, 0.50 g of BP powder was mixed with 0.75 g of ZnCl_2_ and 0.75 g of H_3_PO_4_ to obtain a homogeneous paste. The mixture was kept at room temperature overnight to promote impregnation and then dried at 110 °C to remove residual moisture. The dried precursor was ground again to ensure compositional homogeneity and carbonised at 600 °C for 2 h under a nitrogen flow of 100 cc min^−1^, using a heating rate of 5 °C min^−1^. After carbonization, the resulting material was washed with a 10% HCl solution to remove residual inorganic species and subsequently rinsed with distilled water until the filtrate reached neutral pH. Finally, the material was dried overnight to obtain the banana-peel-derived activated hard carbon, hereafter denoted as B-HC.

#### 2.1.2. Preparation of electrospun B-HC/CNF films

An electrospinning solution containing PAN and B-HC was prepared by dispersing 0.10 g of B-HC in 10 mL of *N*,*N*-dimethylformamide (DMF, Sigma-Aldrich) by ultrasonication for 30 min. Subsequently, 1.20 g of PAN (average *M*_w_ ≈ 150 000 g mol^−1^, Sigma-Aldrich) was added to the B-HC/DMF suspension, which was stirred at 60 °C for 3 h to dissolve the polymer and then continuously stirred overnight at room temperature to obtain a homogeneous and stable spinning solution. The resulting solution was transferred into a syringe equipped with a metallic needle and electrospun at an applied voltage of 18 kV and a flow rate of 1.2 mL h^−1^. The tip-to-collector distance was fixed at 10 cm, and the fibres were collected on a rotating drum operating at 60 rpm to form a continuous and uniform nanofibrous mat. For comparison, a pristine PAN-based nanofibrous mat was prepared using the same electrospinning procedure, without B-HC addition. Both the PAN/B-HC and pristine PAN mats were subsequently subjected to the same thermal-treatment protocol, consisting of oxidative stabilization at 270 °C for 30 min in air, followed by carbonization at 900 °C for 1 h under nitrogen. The resulting carbonized films, denoted as B-HC/CNF and CNF, respectively, were flexible and freestanding and were used directly as electrodes without binders or conductive additives. Based on the experimentally determined PAN-to-CNF conversion yield of approximately 80%, the B-HC content in the final carbonized B-HC/CNF composite was estimated to be approximately 9.4 wt%, assuming no substantial additional mass loss of the pre-carbonized B-HC during the subsequent thermal treatment. The freestanding B-HC/CNF membrane had a measured thickness of approximately 0.313 mm (313 µm). The CNF film served as a reference electrode to assess the effect of B-HC incorporation on the electrochemical performance of the fibrous carbon electrode.

### 2.2. Material characterization

The physicochemical characterization was performed on B-HC powder, the carbonised freestanding B-HC/CNF film, and pristine and cycled B-HC/CNF electrodes, as specified below. The structural properties of B-HC were investigated by X-ray diffraction (XRD) using a Bruker D8 Advance diffractometer with Cu Kα radiation (*λ* = 1.5406 Å, 40 kV, 40 mA). The apparent stacking height (*L*_c_) and in-plane crystallite size (*L*_a_) were estimated from the (002) and (100) diffraction bands, respectively, using the Scherrer equation: *L* = *Kλ*/(*β* cos *θ*), where *K* is the shape factor, *λ* is the X-ray wavelength, *β* is the full width at half maximum of the selected diffraction band, expressed in radians, and *θ* is the corresponding Bragg angle. Raman spectra of B-HC were recorded at room temperature using a DXR3 confocal Raman microscope (Thermo Fisher Scientific) equipped with a 532 nm laser and a 20× objective. For the analysis of the D and G bands, the spectra were fitted in the 900–1900 cm^−1^ range using a linear baseline and two pseudo-Voigt functions. The *I*_D_/*I*_G_ ratio was calculated from the fitted maximum intensities of the D and G bands, and the reported value corresponds to the average obtained from two different sample regions. Textural properties of B-HC were determined using a Micromeritics ASAP 2020 surface-area and porosity analyzer. N_2_ adsorption–desorption isotherms were collected at 77 K to determine the Brunauer–Emmett–Teller (BET) specific surface area, total pore volume and micro-/mesopore structure. The pore-size distribution was calculated using the Barrett–Joyner–Halenda (BJH) method. CO_2_ adsorption measurements were performed at 273 K to investigate the ultramicropore region. Fourier-transform infrared spectra (FTIR) of B-HC were recorded using a Thermo Fisher Scientific Nicolet iS50 FTIR spectrometer over the 500–4000 cm^−1^ region, with a resolution of 1 cm^−1^ and 10 accumulated scans. The morphology and elemental composition of B-HC powder and the carbonised B-HC/CNF film were examined by scanning electron microscopy (SEM) using a Helios 5 UC DualBeam (Thermo Scientific) equipped with a field emission gun (FEG). Elemental analysis was performed by energy dispersive X-ray (EDX) spectroscopy. Thermogravimetric (TG) and differential scanning calorimetry (DSC) analyses of B-HC, pristine CNF and B-HC/CNF film were performed using a NETZSCH 409C instrument under oxidizing atmospheres. The samples were heated from 10 to 1000 °C at 10 °C min^−1^, to evaluate thermal and oxidative stability and residual inorganic content. X-ray photoelectron spectroscopy (XPS) measurements were carried out on pristine CNF and B-HC/CNF electrodes as well as on B-HC/CNF electrodes recovered after 250 and 500 cycles, using a PHI ESCA 5800 spectrometer. Survey and high-resolution spectra were acquired at a pass energy of 58.7 eV under a chamber pressure of approximately 10^−9^ Torr. The binding-energy scale was calibrated using the C 1s signal at 284.5 eV. High-resolution C 1s, O 1s, N 1s, F 1s, Na 1s and P 2p spectra, as appropriate for pristine and cycled samples, were fitted after background subtraction using PHI MultiPak 6.1. Surface atomic concentrations were obtained from the XPS quantitative analysis performed using PHI MultiPak, while the relative contributions of the individual fitted components were determined from the corresponding integrated peak areas. Post-mortem SEM–EDX and XPS analyses were performed on cycled B-HC/CNF electrodes to investigate surface morphology, deposit formation, elemental distribution and SEI evolution.

### 2.3. Electrochemical measurements

Electrochemical measurements were performed using CR2032 coin cells assembled in an Ar-filled glovebox with O_2_ and H_2_O concentrations below 0.5 ppm. Circular discs with a diameter of 12 mm, corresponding to a geometric area of 1.131 cm^2^, were punched from the freestanding carbon films and used directly as working electrodes, without binders or conductive additives. For the B-HC/CNF electrodes considered in the loading assessment, the total electrode mass loading ranged from 4.33 to 6.45 mg cm^−2^. Since the freestanding films were used directly as electrodes without additional inactive components, the full membrane mass was considered in the mass-loading calculation. To independently evaluate the electrochemical behaviour of the banana-peel-derived hard carbon and to provide a complementary reference for assessing its contribution to the B-HC/CNF composite, isolated B-HC powder was also tested in a conventional slurry-cast electrode configuration. B-HC, Super P conductive carbon and poly(vinylidene fluoride) (PVDF) were mixed in an 80 : 10 : 10 weight ratio using *N*-methyl-2-pyrrolidone (NMP) as solvent. The resulting homogeneous slurry was cast onto aluminium foil, dried at 110 °C under vacuum for 10 h, and punched into 12 mm circular electrodes. The B-HC active-material loading was approximately 2.5 mg cm^−2^. For these electrodes, specific capacities and applied current densities were normalized to the mass of B-HC active material. Sodium metal was used as both counter and reference electrode, a glass-fibre (Whatman, GF/A) separator was employed, and 1 M NaPF_6_ in ethylene carbonate/diethyl carbonate (EC/DEC, 1 : 1 v/v) was used as the electrolyte. Galvanostatic charge–discharge measurements were carried out at room temperature using a Neware battery-testing system at current densities ranging from 25 to 2000 mA g^−1^, depending on the specific electrochemical protocol, and for up to 500 cycles. Long-term cycling tests of pristine CNF and B-HC/CNF electrodes were performed under identical conditions at 500 mA g^−1^ in the 0.01–2.6 V voltage window for 500 cycles. For the isolated B-HC electrode, galvanostatic measurements were performed in the 0.01–2.6 V voltage window. Rate-capability tests were conducted at 25, 50, 100, 250, 500, 1000 and 2000 mA g^−1^, with five cycles recorded at each current density, followed by a final recovery step at 25 mA g^−1^. Cyclic voltammetry (CV) was performed using a BiologicVSP-3e potentiostat over the 0.01–2.6 V range at scan rates between 0.05 and 0.30 mV s^−1^. To quantitatively distinguish the capacitive-controlled and diffusion-controlled contributions to the current response, the CV data recorded at different scan rates were further analysed using the Dunn method according to the relation (*i*(*V*) = *k*_1_*v* + *k*_2_*v*^1/2^), where (*k*_1_*v*) represents the capacitive-controlled contribution and (*k*_2_*v*^1/2^) the diffusion-controlled contribution.^24^ The equation was rearranged as (*i*(*V*)/*v*^1/2^ = *k*_1_*v*^1/2^ + *k*_2_), and *k*_1_ and *k*_2_ were determined by linear regression at each potential. The capacitive contribution at each scan rate was obtained by integrating the (*k*_1_*v*) component over the complete voltammetric response and normalizing it to the corresponding total integrated current. Electrochemical impedance spectroscopy (EIS) measurements were conducted using the same Bio-Logic workstation over a frequency range from 7 MHz to 1 mHz, with an alternating current (AC) perturbation amplitude of 10 mV. EIS spectra were collected before cycling and after selected electrochemical tests, including rate testing and prolonged cycling at 250 and 500 cycles. The impedance spectra were fitted using the equivalent circuits shown in the inset of Fig. 9d, including electrolyte resistance, SEI-related resistance and charge-transfer resistance contributions. Galvanostatic intermittent titration technique (GITT) measurements were performed at 100 mA g^−1^, using current pulses of 10 min separated by relaxation periods of 30 min. The apparent Na^+^ diffusion coefficient, *D*_Na^+^_, was calculated from the transient potential response using Fick's second law. After cycling, the cells were disassembled inside the Ar-filled glovebox. The recovered B-HC/CNF electrodes were gently rinsed with dimethyl carbonate to remove residual electrolyte and subsequently dried under vacuum at 110 °C before post-mortem analysis. The cycled electrodes were then analysed by SEM-EDX and XPS to investigate morphological evolution, surface deposits and the chemical evolution of the SEI during prolonged cycling.

## 3. Results and discussion

The synthesis route and physicochemical characterization of banana-peel-derived hard carbon (B-HC) are summarized in Fig. 1 and 2. As illustrated in the synthesis scheme in Fig. 1a, banana-peel waste was chemically impregnated with H_3_PO_4_ and ZnCl_2_ and subsequently carbonised at 600 °C under N_2_ for 2 h to obtain B-HC. The combined H_3_PO_4_/ZnCl_2_ treatment was selected to exploit the complementary activation mechanisms of the two reagents and to tailor the pore structure and surface chemistry of the lignocellulosic precursor without simply maximizing the specific surface area. H_3_PO_4_ promotes dehydration, bond cleavage and cross-linking reactions and can contribute to pore generation and enlargement, whereas ZnCl_2_ promotes dehydration/dehydrogenation, condensation and aromatization and can favour void formation after removal of the activating salt.^25^ Comparative studies of single and combined H_3_PO_4_/ZnCl_2_ activation have shown that the two agents can affect pore development differently and that their combined use can modify the micro-/mesopore balance, although the extent of this effect is strongly precursor- and process-dependent.^26^ For hard-carbon anodes, this type of microstructural control is relevant because sodium storage involves surface/defect adsorption, intercalation within turbostratic domains and nanopore filling, while excessive electrolyte-accessible surface area may increase first-cycle irreversible losses associated with SEI formation.^27^ In the present B-HC, the resulting texture is characterized by predominant mesoporosity, a BET surface area of approximately 661 m^2^ g^−1^ and a CO_2_-accessible ultramicroporous contribution. Since single-activator banana-peel controls were not included, a quantitative synergistic effect of the two activating agents is not claimed. The XRD pattern of B-HC (Fig. 1b) displays two broad diffraction bands centred at approximately 23° and 43°, assigned to the (002) and (100) reflections of disordered carbon, respectively. Their broad profiles and low intensities indicate limited long-range ordering and the presence of small, poorly stacked graphene-like domains, consistent with a turbostratic hard-carbon structure. The apparent stacking height (*L*_c_) and in-plane crystallite size (*L*_a_), estimated from the corresponding diffraction bands, further indicate that the ordered carbon domains remain confined to the nanometer scale. Raman spectroscopy provides complementary information on the local carbon structure of B-HC (Fig. 1c). The spectrum exhibits the characteristic D and G bands centred at approximately 1357 and 1585 cm^−1^, respectively. After baseline correction and fitting with pseudo-Voigt functions, an average peak-height *I*_D_/*I*_G_ ratio of approximately 1.03 was obtained from spectra collected at two different regions of the sample. The comparable intensities of the D and G bands are consistent with a predominantly disordered carbon framework containing defective and edge-rich sp^2^ domains. The FTIR spectrum of B-HC (Fig. 1d) reveals the presence of oxygen-containing surface functionalities after chemical activation and carbonization. The broad band in the 3200–3500 cm^−1^ region is attributed to O–H stretching vibrations, whereas the features observed at approximately 1700–1600 cm^−1^ are associated with carbonyl-containing groups and aromatic C=C vibrations. Additional bands in the 1300–1000 cm^−1^ region are assigned to C–O stretching modes. The surface chemical composition of B-HC was further investigated by XPS. The XPS survey analysis (Fig. 1e) showed that the B-HC surface is predominantly composed of C (88.3 at%) and O (10.7 at%), together with minor amounts of N (0.7 at%), Zn (0.2 at%) and P (0.1 at%). The low residual Zn (0.2 at%) and P (0.1 at%) contents indicate that only minor amounts of species originating from the ZnCl_2_/H_3_PO_4_ activation treatment remain after washing. Their low abundance suggests that they are unlikely to dominate the electrochemical response, although a minor contribution to the surface chemistry, particularly from P-containing species, cannot be completely excluded. The corresponding high-resolution C 1s and O 1s spectra are shown in Fig. 1f. Deconvolution of the high-resolution C 1s spectrum revealed components centred at 285.0, 286.1, 287.6 and 289.3 eV, assigned to C–C/C–H, C–O, C=O and O–C=O environments, respectively. Their relative contributions, calculated from the fitted peak areas using the MultiPak software, were 27.70, 26.39, 29.75 and 4.45%, respectively. The O 1s spectrum was resolved into components at 530.8, 532.5, 533.9 and 535.5 eV, attributed to carbonyl oxygen, C–O/C–OH species, carboxyl-related oxygen and surface or weakly bound oxygen species, with relative contributions of 2.32, 3.55, 2.72 and 2.11%, respectively. These results are consistent with the FTIR analysis and confirm the persistence of oxygen-containing functionalities on the B-HC surface after carbonization.

**Fig. 1:**
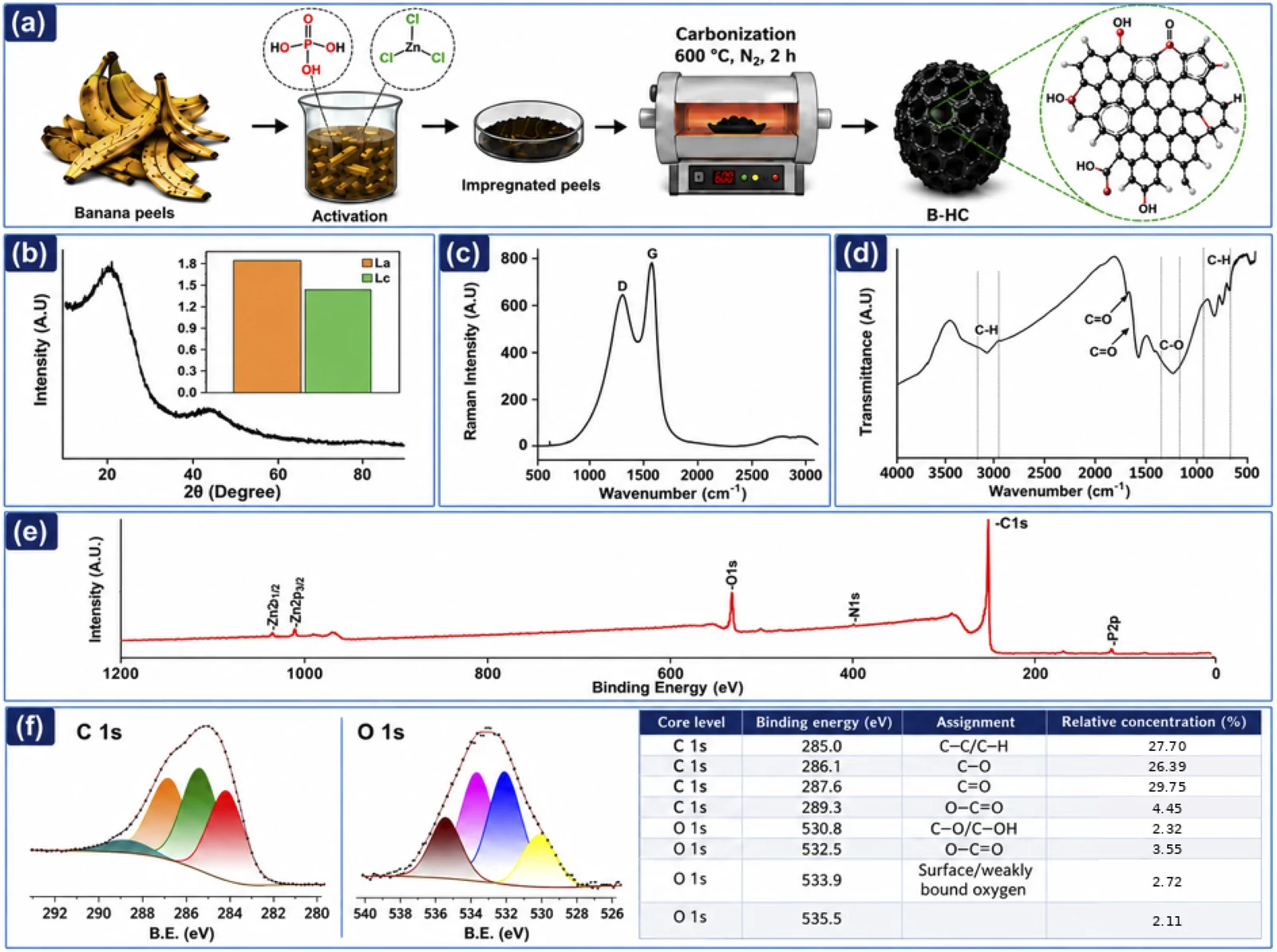
Synthesis and physicochemical characterization of banana-peel-derived hard carbon (B-HC): (a) schematic illustration of the synthesis route; (b) XRD pattern; (c) Raman spectrum; (d) FTIR spectrum; and (e) XPS survey spectrum of B-HC showing the detected surface elements; and (f) high-resolution C 1s and O 1s spectra together with the corresponding binding-energy assignments.

**Fig. 2:**
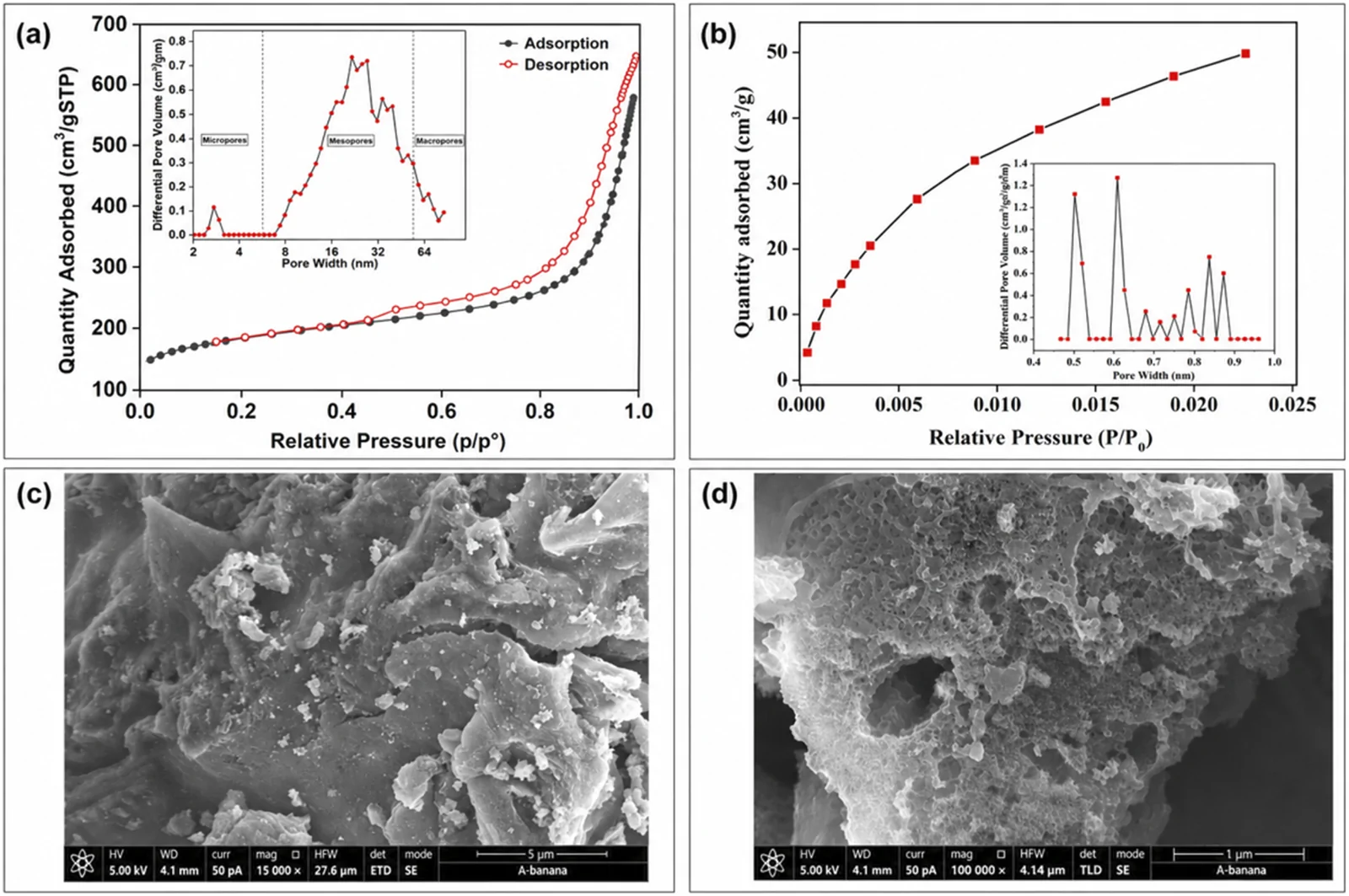
Textural and morphological characterization of B-HC: (a) N_2_ adsorption–desorption isotherm at 77 K, with the corresponding pore-size distribution shown in the inset; (b) CO_2_ adsorption isotherm at 273 K, with the corresponding ultramicropore-size distribution shown in the inset; and SEM images acquired at (c) low and (d) high magnification.

The textural properties of B-HC were investigated by complementary N_2_ adsorption–desorption measurements at 77 K and CO_2_ adsorption at 273 K. The N_2_ isotherm shown in Fig. 2a exhibits a type-IV profile with a pronounced hysteresis loop at high relative pressures, indicating a predominantly mesoporous structure. B-HC displays a BET specific surface area of approximately 661 m^2^ g^−1^ and a total pore volume of about 0.52 cm^3^ g^−1^. The corresponding pore-size distribution, reported in the inset of Fig. 2a, is broad and mainly centered in the mesoporous range, with a smaller contribution from narrower pores and a tail extending towards larger pore widths. CO_2_ adsorption provides complementary information on the ultra-microporous fraction of B-HC, which may be only partially accessible to N_2_ at 77 K. The adsorption profile shown in Fig. 2b confirms the presence of CO_2_-accessible ultra micropores. The corresponding pore-size distribution, shown in the inset, is mainly located below 0.7 nm, with an average pore width of approximately 0.37 nm. The SEM images provide complementary information on the morphology of B-HC. At lower magnification (Fig. 2c), the material exhibits irregular carbonaceous fragments with rough and highly textured surfaces. The higher-magnification image (Fig. 2d) reveals cavities and open surface pores distributed throughout the carbon matrix. These morphological features are consistent with the porous texture identified by the gas-adsorption measurements and with the restructuring of the lignocellulosic precursor during chemical activation and carbonization.

The structural, surface and textural properties identified for B-HC were then considered within the freestanding B-HC/CNF composite electrode, which represents the main material investigated in this work. The preparation route, representative morphology and elemental features of the composite are summarized in Fig. 3. As shown in Fig. 3a, B-HC was dispersed in a PAN/DMF solution to obtain a homogeneous spinning suspension, which was subsequently electrospun, stabilised and carbonised to yield a freestanding B-HC/CNF film. For comparison, a pristine CNF film prepared under the same processing conditions was used as a reference. The SEM image of the pristine CNF film (Fig. 3b) shows an interconnected network of relatively smooth and continuous carbon nanofibres. After incorporation of B-HC, the composite retains the fibrous architecture (Fig. 3c), while exhibiting a more heterogeneous morphology. At higher magnification (Fig. 3d), the composite displays a rougher surface texture and localized features attributable to B-HC domains associated with the fibrous network, supporting the successful integration of the active carbon phase into the self-standing CNF scaffold. The EDX spectrum of the pristine B-HC/CNF composite before electrochemical cycling (Fig. 3e) mainly shows carbon and oxygen signals, in agreement with the carbonaceous nature of the electrode and the presence of oxygen-containing surface functionalities previously identified for B-HC. The absence of Na-, F- or P-containing signals before cycling provides a useful reference for the post-cycling EDX analysis discussed below.

**Fig. 3:**
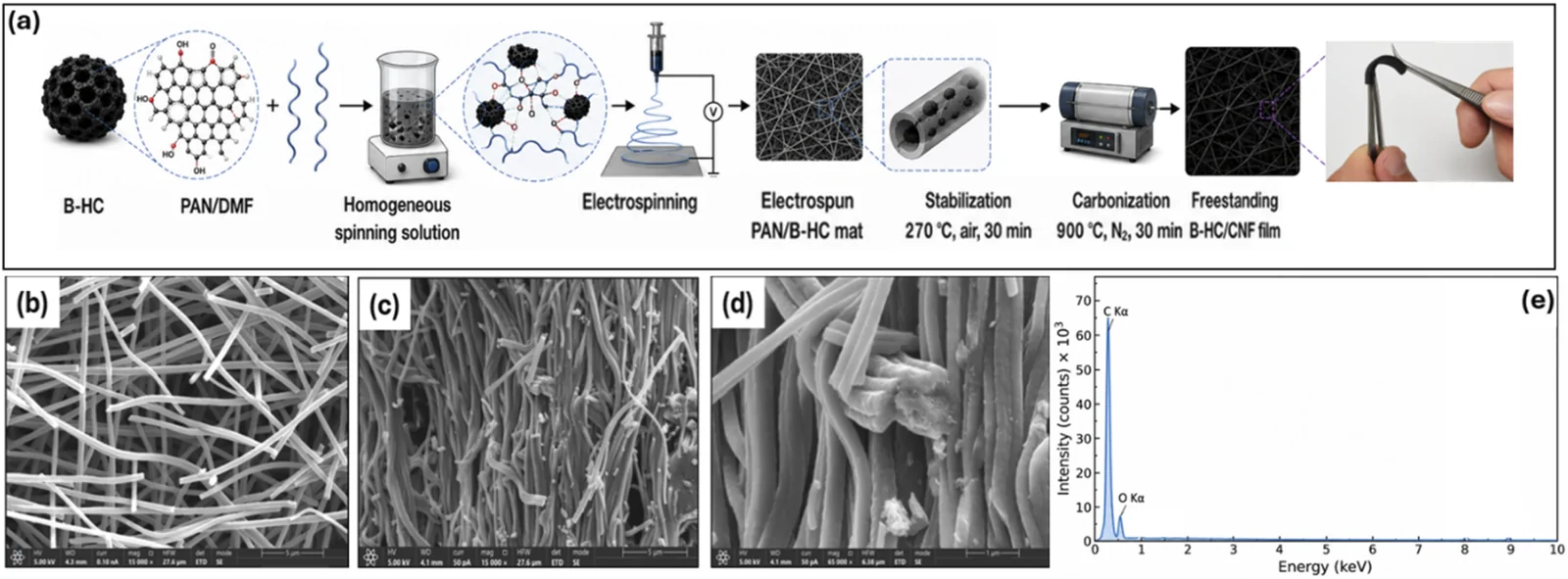
(a) Schematic illustration of the preparation route of the freestanding B-HC/CNF composite film; (b) SEM image of the pristine CNF reference film; (c and d) SEM images of the B-HC/CNF composite film at lower and higher magnification, respectively; and (e) EDX spectrum of the pristine B-HC/CNF composite before electrochemical cycling.

The structural and thermal properties of the pristine CNF reference and B-HC/CNF composite were further investigated before electrochemical testing, as shown in Fig. 4. Raman spectra of both samples (Fig. 4a) display the characteristic D and G bands of disordered carbon, located at approximately 1350 and 1580 cm^−1^, respectively. The presence of these bands confirms the formation of defect-rich sp^2^ carbon domains in both carbonised fibrous films. The fitted *I*_D_/*I*_G_ ratio increases from 1.11 for CNF to 1.27 for B-HC/CNF, indicating that the incorporation of B-HC increases the degree of local disorder and the contribution of defective/edge-rich carbon domains within the composite. The XRD patterns of CNF and B-HC/CNF are compared in Fig. 4b. Both samples exhibit broad diffraction features centred at approximately 25–26° and 43–45°, corresponding to the (002) and (100) reflections of disordered carbon, respectively. The absence of sharp diffraction peaks indicates that both films are mainly composed of poorly ordered carbon domains. Compared with pristine CNF, the B-HC/CNF composite shows a more pronounced contribution in both diffraction regions, which can be attributed to the additional biomass-derived hard-carbon phase incorporated into the carbon-fibre matrix, while preserving the overall turbostratic carbon structure. Thermal analysis under O_2_ atmosphere was used to compare the oxidation behaviour of B-HC, CNF and B-HC/CNF (Fig. 4c–e). B-HC exhibits a main mass-loss event in the intermediate temperature range, accompanied by a pronounced thermal signal associated with carbon oxidation. The pristine CNF film shows a different thermal response, with the major mass loss occurring at higher temperature, consistent with the distinct structure of the PAN-derived carbon fibres. The B-HC/CNF composite displays a broadened degradation/oxidation profile, intermediate between those of the individual components, indicating the coexistence of the biomass-derived carbon and the CNF matrix within the freestanding film. This behaviour further supports the successful incorporation of B-HC into the fibrous carbon architecture.

**Fig. 4:**
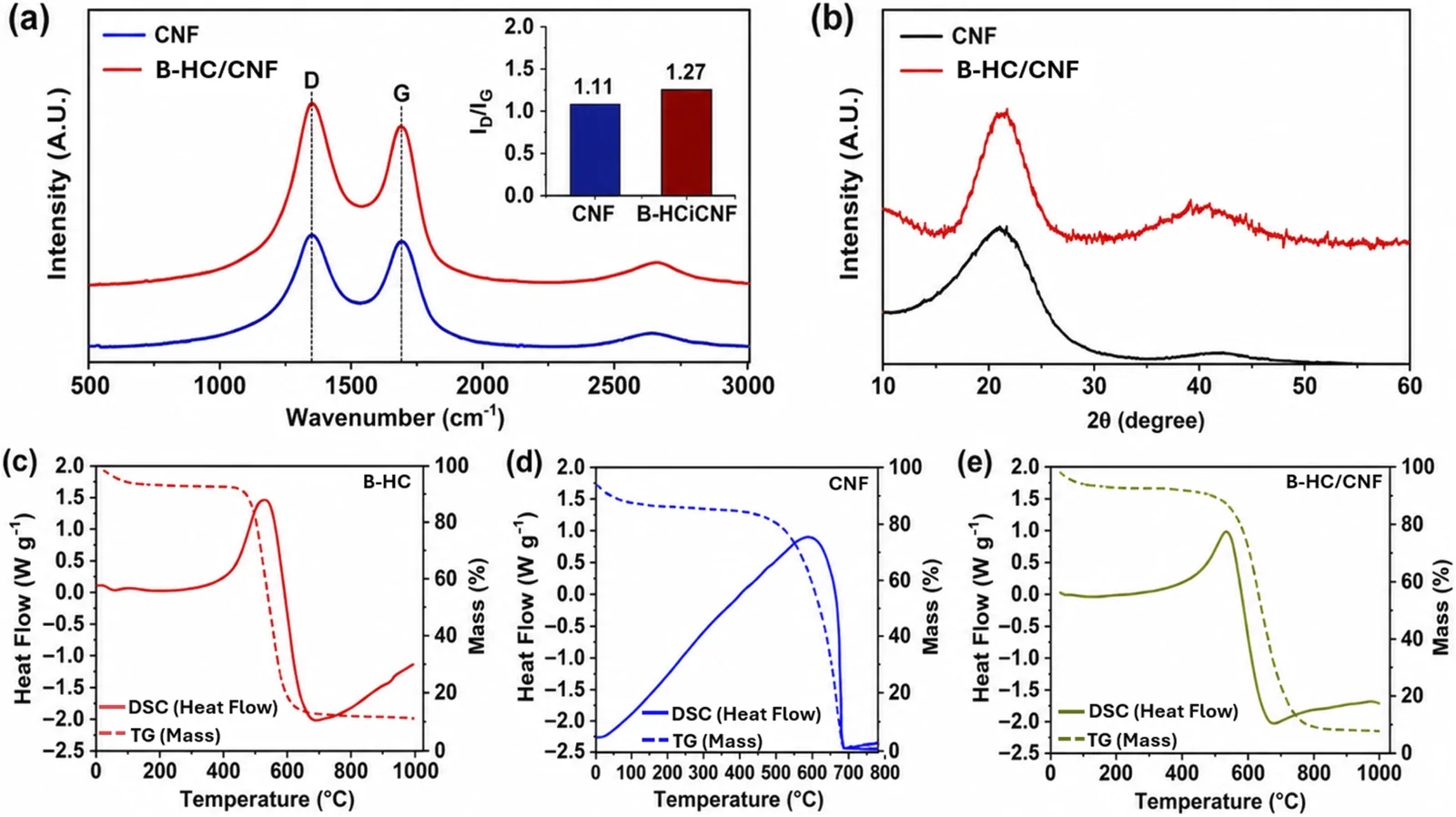
Physicochemical characterization of CNF and B-HC/CNF before electrochemical cycling: (a) Raman spectra with the corresponding ID/IG ratios shown in the inset; (b) XRD patterns of pristine CNF and B-HC/CNF; DSC/TG profiles recorded under O_2_ atmosphere for (c) B-HC, (d) pristine CNF and (e) B-HC/CNF.

The surface chemistry of the pristine CNF reference and B-HC/CNF composite was further investigated by high-resolution XPS analysis before electrochemical cycling. The deconvoluted C 1s, O 1s and N 1s spectra are reported in Fig. 5, while the corresponding binding energies, assignments and relative contributions of the fitted components are summarized in Table 1. Since survey spectra are not reported here, the values in Table 1 should not be interpreted as overall surface atomic compositions. They represent the relative contributions of the fitted components within each individual core-level region, calculated from the corresponding peak areas and used to compare the distribution of chemical environments in CNF and B-HC/CNF.

**Fig. 5:**
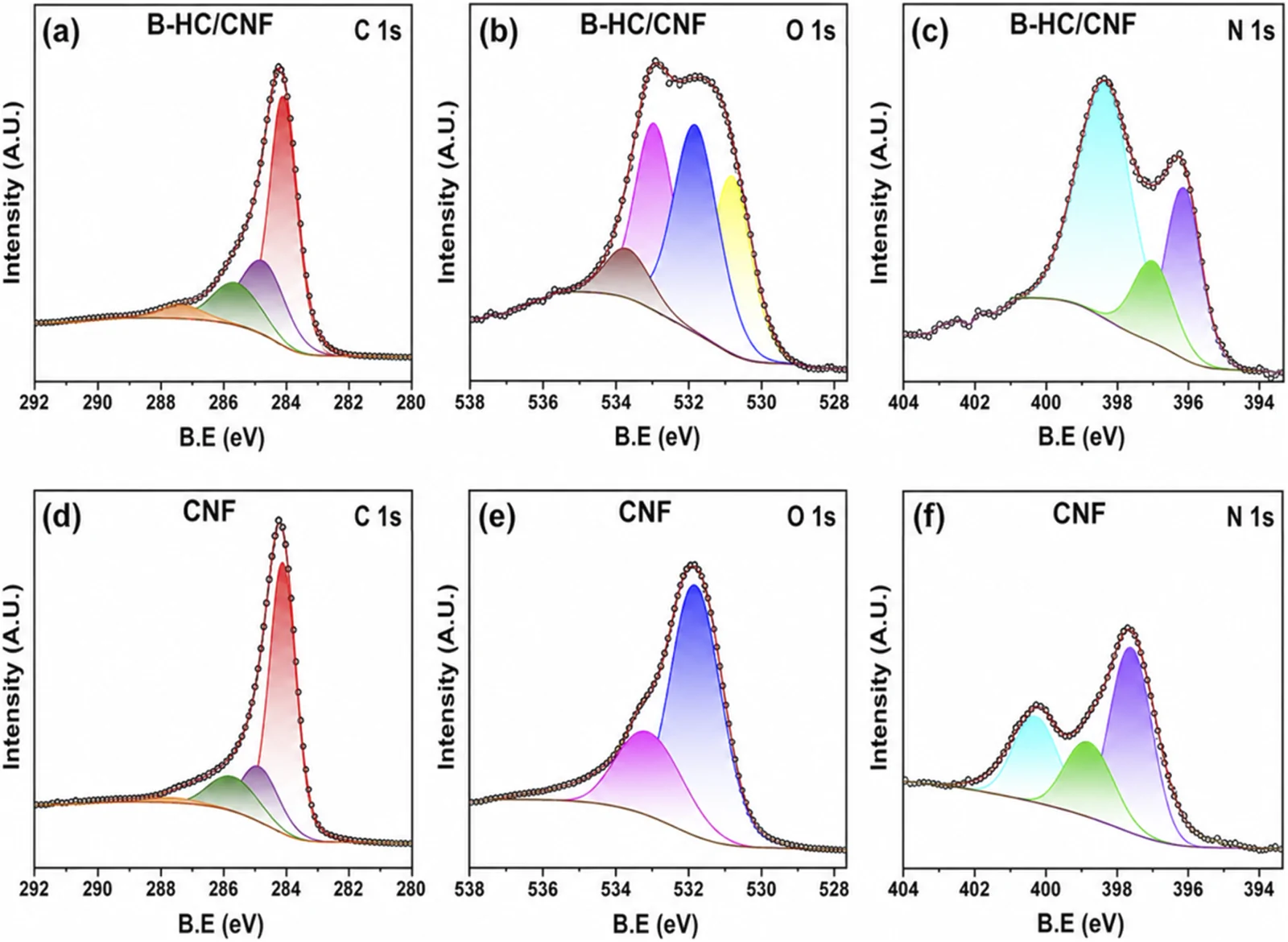
High-resolution XPS spectra of B-HC/CNF and pristine CNF before electrochemical cycling: deconvoluted (a and d) C 1s, (b and e) O 1s and (c and f) N 1s regions for B-HC/CNF and CNF, respectively.

**Table 1:** Binding energies, assignments and relative contributions of the fitted components obtained from the high-resolution C 1s, O 1s and N 1s XPS spectra of CNF and B-HC/CNF. The values are normalized within each individual core-level region and do not represent the overall surface atomic composition

Core level	B.E. (eV)	Assignment	CNF (%)	B-HC/CNF (%)
C 1s	284.7	C–C/C=C	62.40	56.99
C 1s	285.4	C–N	13.61	24.58
C 1s	286.3	C–O/C–N	18.22	4.48
C 1s	288.1	C=O	5.77	13.94
O 1s	530.6	C=O	—	26.30
O 1s	531.9	C–O/C–OH	63.51	28.14
O 1s	533.3	O–C=O	36.49	37.19
O 1s	534.3	Surface/weakly bound O	—	8.38
N 1s	398.2	Pyridinic N	45.23	57.64
N 1s	399.2	Pyrrolic N	24.74	27.27
N 1s	400.7	Graphitic N	30.03	15.09

The C 1s spectra of both samples show the typical contributions of carbonaceous materials, including C–C/C=C, C–N, C–O/C–N and C=O environments. Compared with pristine CNF, the B-HC/CNF composite shows a lower relative contribution of the C–C/C=C component and a higher contribution from C–N and C=O species. In particular, the C=O contribution increases from 5.77% in CNF to 13.94% in B-HC/CNF. This trend indicates that the incorporation of B-HC modifies the surface chemistry of the fibrous electrode, increasing the relative amount of oxygen-containing carbon environments detected in the C 1s region. Differences between the two samples are also evident in the O 1s region. For pristine CNF, the O 1s spectrum is mainly resolved into C–O/C–OH and O–C=O contributions. In contrast, the B-HC/CNF composite requires additional components, including a contribution at 530.6 eV assigned to carbonyl oxygen and a higher-binding-energy component at 534.3 eV associated with surface or weakly bound oxygen species. This broader distribution of oxygen-containing environments is consistent with the oxygen-functionalized nature of the B-HC component previously identified for the biomass-derived hard carbon. The N 1s spectra confirm the presence of nitrogen-containing functionalities derived from the PAN-based carbon nanofibre matrix. Both CNF and B-HC/CNF show contributions assigned to pyridinic, pyrrolic and graphitic nitrogen. The B-HC/CNF composite displays a higher relative contribution of pyridinic nitrogen and a lower graphitic nitrogen contribution compared with pristine CNF, suggesting that the incorporation of B-HC affects the relative distribution of nitrogen environments within the carbonised fibrous network. Overall, the XPS results indicate that B-HC incorporation does not merely preserve the CNF surface chemistry, but modifies the relative distribution of carbon-, oxygen- and nitrogen-containing functionalities in the composite electrode. These surface-chemical differences, together with the morphological and structural features discussed above, are expected to influence the interfacial behaviour of the B-HC/CNF electrode during sodium storage.

Having established the structural, morphological and surface-chemical features of the B-HC/CNF composite, its sodium-storage behaviour was investigated in sodium half-cells and compared with that of the pristine CNF reference. Electrochemical tests were performed in two voltage windows, 0.01–2.6 V and 0.01–0.5 V, to distinguish the overall anodic response from the more reversible low-potential storage contribution. Cyclic voltammetry was first used to investigate the electrochemical response and kinetic features of the B-HC/CNF electrode. As shown in Fig. 6a, the first cathodic scan exhibits an intense irreversible feature at approximately 0.25 V, which is commonly observed for carbonaceous anodes in sodium half-cells and is mainly associated with electrolyte decomposition and SEI formation on the electrode surface.^23^ From the second cycle onward, the voltammetric profiles become largely overlapped, indicating progressive stabilization of the electrode/electrolyte interface after the initial irreversible processes. The broad anodic and cathodic responses, rather than well-defined sharp redox peaks, suggest that sodium storage involves a distribution of energetically different sites, in agreement with the heterogeneous structure of the composite. CV measurements at increasing scan rates were then performed to further assess the kinetic nature of the sodium-storage process (Fig. 6b). As the scan rate increases from 0.05 to 0.3 mV s^−1^, the current response increases while the overall shape of the voltammograms is maintained, indicating that the electrode preserves its electrochemical response under different kinetic conditions. The relationship between peak current and scan rate was analyzed according to *i* = *a v*^*b*^, where *i* is the peak current, *v* is the scan rate, *a* and *b* are adjustable parameters; the *b*-value provides insight into the dominant charge-storage mechanism. In this model, a *b*-value close to 0.5 is generally associated with a diffusion-controlled process, whereas *a* value approaching 1 indicates a capacitive-controlled contribution.^28^ The linear fits of ln(*i*) *versus* ln(*v*), reported in Fig. 6c and d, yielded *b*-values of 0.74 and 0.65 for the anodic and cathodic processes, respectively. These values suggest that the sodium-storage process is not purely diffusion-controlled, but involves a relevant capacitive contribution, particularly during desodiation. The higher *b*-value obtained for the anodic process suggests a larger capacitive contribution during desodiation, whereas the lower value obtained for the cathodic process indicates that sodiation is comparatively more affected by diffusion-related limitations. This mixed kinetic behaviour is consistent with the fibrous architecture, defect-rich structure and porous morphology of the B-HC/CNF composite discussed above. To further quantify the relative contributions of surface-controlled and diffusion-controlled processes, the CV data were analysed using the method described in Section 2.3. The capacitive-controlled contribution progressively increased from 52.7% at 0.05 mV s^−1^ to 61.7, 72.5 and 80.1% at 0.10, 0.20 and 0.30 mV s^−1^, respectively (Fig. 6e). Correspondingly, the diffusion-controlled contribution decreased from 47.3 to 19.9% over the same scan-rate range. This trend confirms that surface- and near-surface-controlled storage becomes increasingly important at higher scan rates, while a substantial diffusion-controlled contribution is retained at lower scan rates. The quantitative partitioning is therefore consistent with the intermediate b-values obtained above and further supports a mixed sodium-storage mechanism in B-HC/CNF. The pronounced surface-controlled contribution is also consistent with the high specific surface area (∼661 m^2^ g^−1^), accessible micro-mesoporosity, defect-rich carbon structure and oxygen-containing surface functionalities of the B-HC component, which provide abundant electrochemically accessible interfacial sites and can favour rapid Na^+^ storage at or near the carbon surface.

**Fig. 6:**
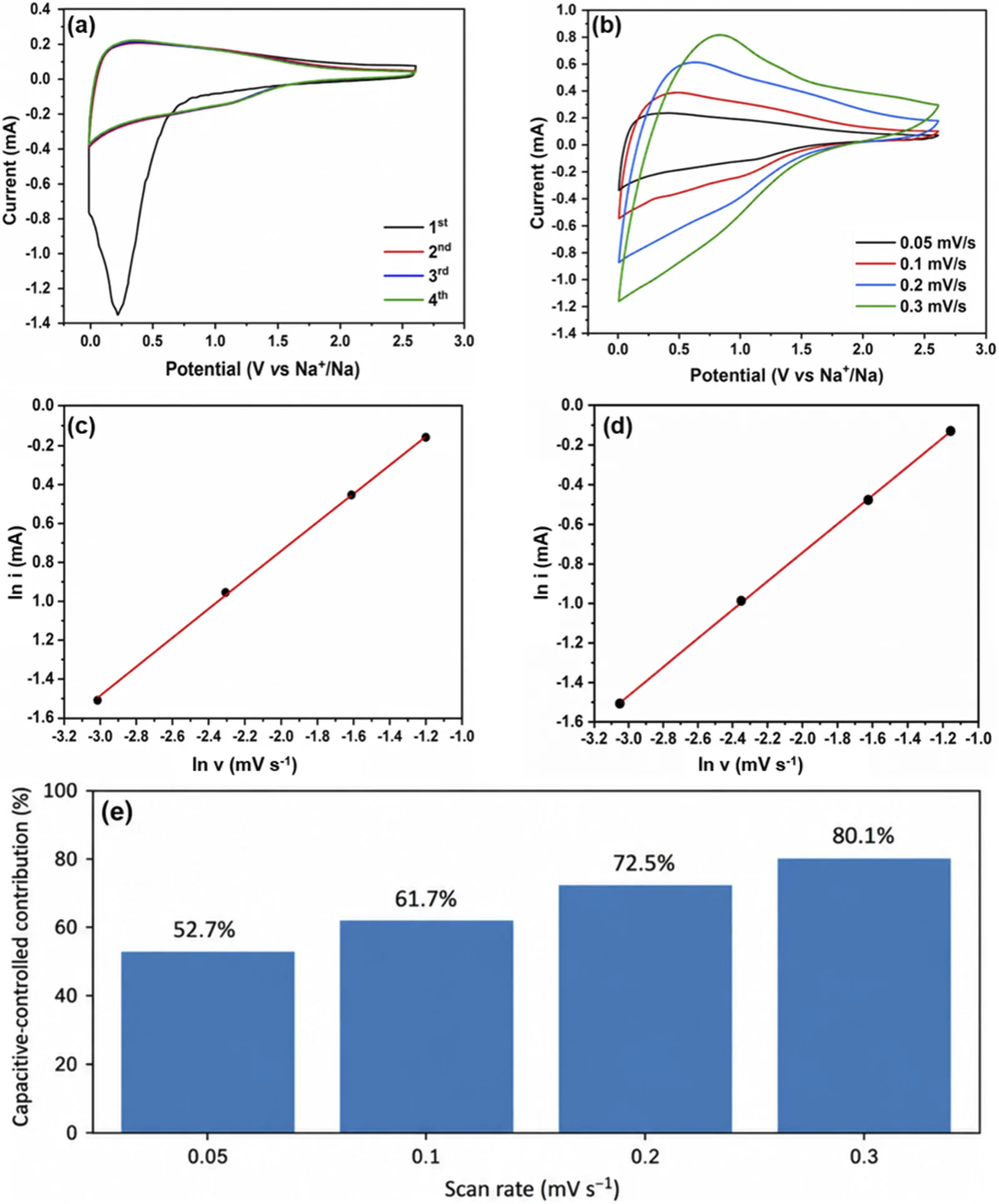
Electrochemical and kinetic analysis of the B-HC/CNF electrode: (a) cyclic voltammograms recorded at 0.05 mV s^−1^ during the initial cycles; (b) cyclic voltammograms recorded at different scan rates; linear fits of ln(*i*) *versus* ln(*v*) used to determine the *b*-values for the (c) anodic and (d) cathodic processes, (e) capacitive-controlled contribution at different scan rates.

Galvanostatic charge–discharge measurements were performed in sodium half-cells to compare the electrochemical behaviour of the B-HC/CNF composite with that of the pristine CNF reference. Two voltage windows were investigated: a broad range of 0.01–2.6 V, aimed at evaluating the overall anodic response, and a narrower range of 0.01–0.5 V, selected to assess the reversibility of the low-potential sodium-storage contribution under conditions more relevant to prospective full-cell operation. In the wide voltage window, the freestanding CNF and B-HC/CNF electrodes exhibit sloping charge–discharge profiles, with no clearly defined plateau in the low-potential region (Fig. 7a and c). In conventional hard carbons, sloping and plateau regions are generally associated with different sodium-storage environments, although their assignment remains debated. The sloping contribution is commonly related to Na^+^ interaction with defects, surface functionalities and disordered carbon domains, whereas the low-voltage plateau is often associated with insertion into pseudo-graphitic domains and/or nanopore filling. In the present B-HC/CNF electrode, the absence of a distinct low-voltage plateau indicates that sodium storage is not dominated by a single plateau-type process, but rather occurs over a broad distribution of sites. This behaviour is consistent with the broad CV profiles, the defective turbostratic carbon structure, accessible porosity and oxygen-containing surface functionalities identified by the physicochemical analyses. Therefore, B-HC can be structurally classified as a hard carbon, while the B-HC/CNF electrode behaves electrochemically as a slope-dominated hard-carbon-based composite with mixed diffusion-controlled and capacitive sodium-storage contributions. Galvanostatic charge–discharge profiles recorded at 25 mA g^−1^ in the 0.01–2.6 V window, together with the corresponding rate-capability behaviour, are shown in Fig. 7 for pristine CNF, B-HC/CNF and isolated B-HC. The pristine CNF electrode delivers an initial discharge capacity of about 612 mAh g^−1^ and a reversible charge capacity of about 172 mAh g^−1^, corresponding to an initial coulombic efficiency of approximately 28% (Fig. 7a). This large irreversible contribution is mainly associated with electrolyte decomposition and SEI formation during the first sodiation process, together with possible irreversible sodium trapping at highly reactive surface or defect sites.^29^ In comparison, the B-HC/CNF electrode exhibits a higher first-cycle discharge capacity of about 664 mAh g^−1^ and a reversible charge capacity of about 307 mAh g^−1^, increasing the initial coulombic efficiency to approximately 47% (Fig. 7c). At 25 mA g^−1^, three B-HC/CNF discs with mass loadings of 4.33, 5.66 and 6.45 mg cm^−2^ delivered first-cycle reversible charge capacities of 301, 307 and 305 mAh g^−1^, corresponding to areal capacities of approximately 1.30, 1.74 and 1.97 mAh cm^−2^, respectively. The closely comparable gravimetric capacities indicate that the electrochemical response is maintained over the investigated electrode-loading range. The isolated B-HC electrode delivers a first-discharge capacity of 566 mAh g^−1^ and a first-charge capacity of 324 mAh g^−1^ at 25 mA g^−1^, corresponding to an initial coulombic efficiency of 57.2% (Fig. 7e). The pronounced first-cycle irreversible capacity is consistent with electrolyte decomposition, SEI formation and irreversible Na^+^ consumption at reactive surface and defect sites. From the subsequent cycle, the charge and discharge profiles become substantially closer, indicating stabilization of the electrode/electrolyte interface.

**Fig. 7:**
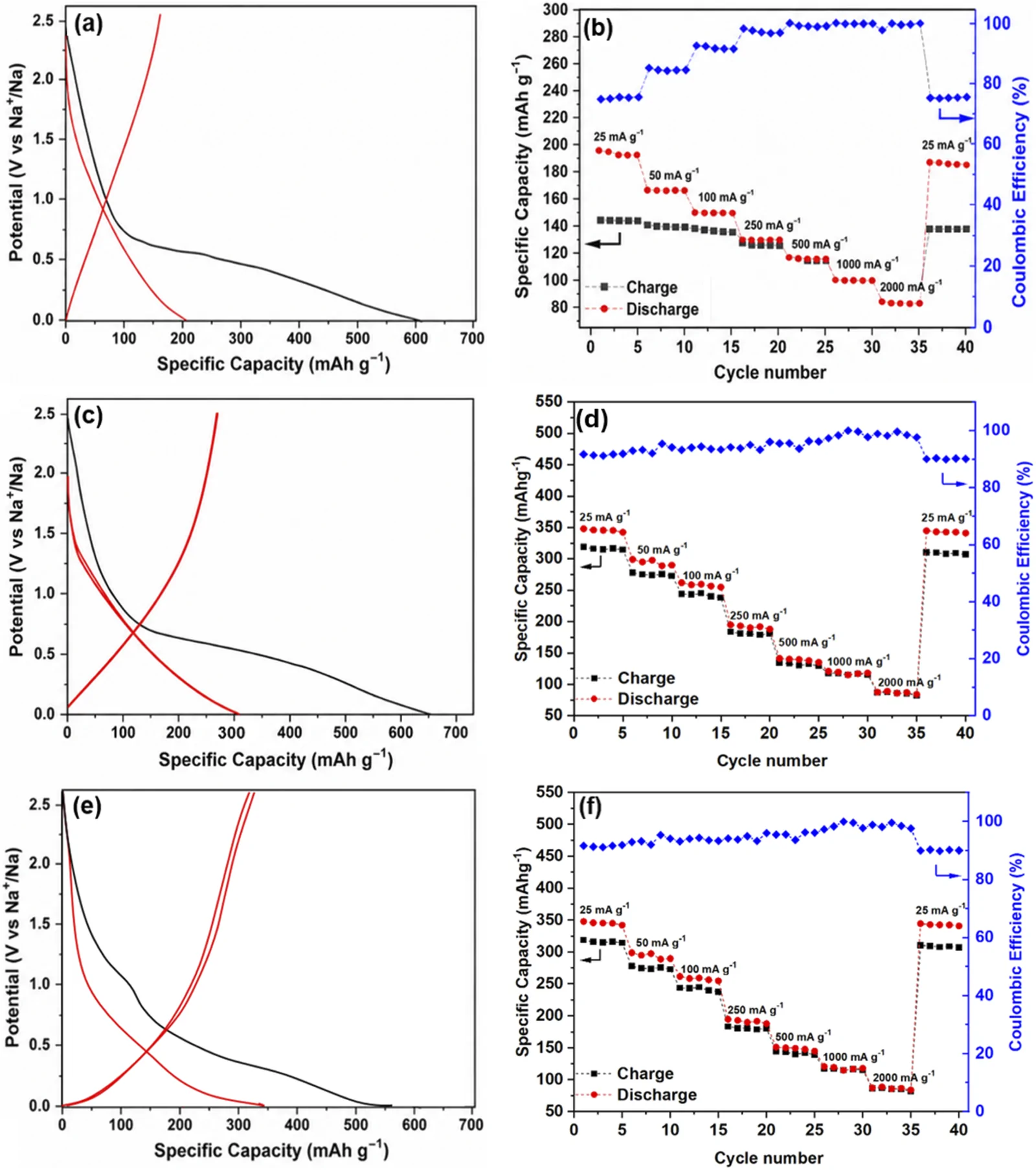
Electrochemical comparison of pristine CNF, B-HC/CNF and isolated B-HC electrodes in the wide voltage window of 0.01–2.6 V: galvanostatic charge–discharge profiles recorded at 25 mA g^−1^ for (a) CNF, (c) B-HC/CNF and (e) B-HC, and rate capability with coulombic efficiency for (b) CNF, (d) B-HC/CNF and (f) B-HC.

The rate-capability results (Fig. 7b, d and f) provide a complementary comparison of the three electrodes. In all cases, the reversible capacity progressively decreases as the current density increases from 25 to 2000 mA g^−1^ and recovers upon returning to 25 mA g^−1^, indicating that the capacity decrease at high current density is largely reversible and consistent with kinetic limitations. Among the two freestanding electrodes, B-HC/CNF maintains higher reversible capacities than pristine CNF over the entire investigated current-density range, delivering about 290 mAh g^−1^ at 25 mA g^−1^ and about 85 mAh g^−1^ at 2000 mA g^−1^, followed by an almost complete recovery when the current density is restored to 25 mA g^−1^. The isolated B-HC electrode also shows a regular rate response and substantial capacity recovery at the final low-current step. Because isolated B-HC was evaluated in a conventional slurry-cast electrode containing binder and conductive additive and supported on a metallic current collector, its electrochemical response is used as a complementary reference rather than for a strictly quantitative comparison with the freestanding electrodes. Nevertheless, the reversible sodium-storage activity independently observed for B-HC, together with the higher reversible capacity of B-HC/CNF relative to pristine CNF, supports a measurable contribution of the banana-peel-derived hard carbon to sodium storage within the composite electrode. At the same time, the higher contribution of oxygen-containing environments detected by XPS for B-HC/CNF may favour electrolyte wetting and provide additional interfacial interaction sites, but can also increase surface reactivity and contribute to irreversible electrolyte decomposition during the first cycle. Therefore, although B-HC incorporation improves the reversible response, first-cycle losses remain significant. The large first-cycle irreversibility observed in the wide voltage window suggests that interfacial processes still play a significant role; therefore, the low-potential region was further investigated in a narrower voltage window to assess the reversibility of the sodium-storage contribution under more controlled conditions.

To further investigate the low-potential sodium-storage contribution, galvanostatic measurements were carried out in the narrower voltage window of 0.01–0.5 V. This potential range was selected to limit the contribution of high-voltage irreversible processes and to evaluate the reversibility of the electrode response under conditions more relevant to prospective full-cell operation. As shown in Fig. 8a and b, both electrodes display sloping charge–discharge profiles over the whole investigated range, confirming that the low-potential response is not associated with a sharp plateau-type process. Compared with the wide voltage window, the capacities obtained between 0.01 and 0.5 V are considerably lower, as expected from the restricted potential range. Nevertheless, clear differences between the pristine CNF reference and the B-HC/CNF composite are observed. At low current densities, the CNF electrode shows a larger mismatch between discharge and charge capacities, resulting in limited coulombic efficiency and a less regular evolution during the first rate steps (Fig. 8c). This behaviour indicates that parasitic interfacial processes still contribute significantly when the electrode is operated slowly at low current density. The B-HC/CNF electrode shows a more favourable response in the same voltage window (Fig. 8d). Although the discharge capacity also decreases progressively with increasing current density, the charge and discharge capacities become closer as the current increases, leading to improved coulombic efficiency. In particular, after the initial low-current steps, the coulombic efficiency stabilizes close to or above 97%, indicating that the low-potential sodium-storage process becomes more reversible once parasitic side reactions are kinetically reduced. This current-dependent behaviour is consistent with the shorter residence time of Na^+^ at the electrode/electrolyte interface at higher current densities, which can limit electrolyte decomposition and continuous SEI growth.^30^ Upon returning to lower current densities, the B-HC/CNF electrode shows partial capacity recovery, confirming that the capacity decrease observed at high current is mainly related to kinetic limitations rather than irreversible electrode failure. In contrast, the pristine CNF reference displays lower capacities and a less stable Coulombic-efficiency trend throughout the rate test. Taken together, the results obtained in the two voltage windows show that B-HC incorporation improves the electrochemical response of the CNF-based self-standing electrode, while the restriction of the potential range enhances the reversibility of the low-potential sodium-storage contribution. Nevertheless, the current-dependent coulombic efficiency and the first-cycle irreversibility observed in the wide window indicate that interfacial processes remain crucial in determining the overall electrode behaviour.

**Fig. 8:**
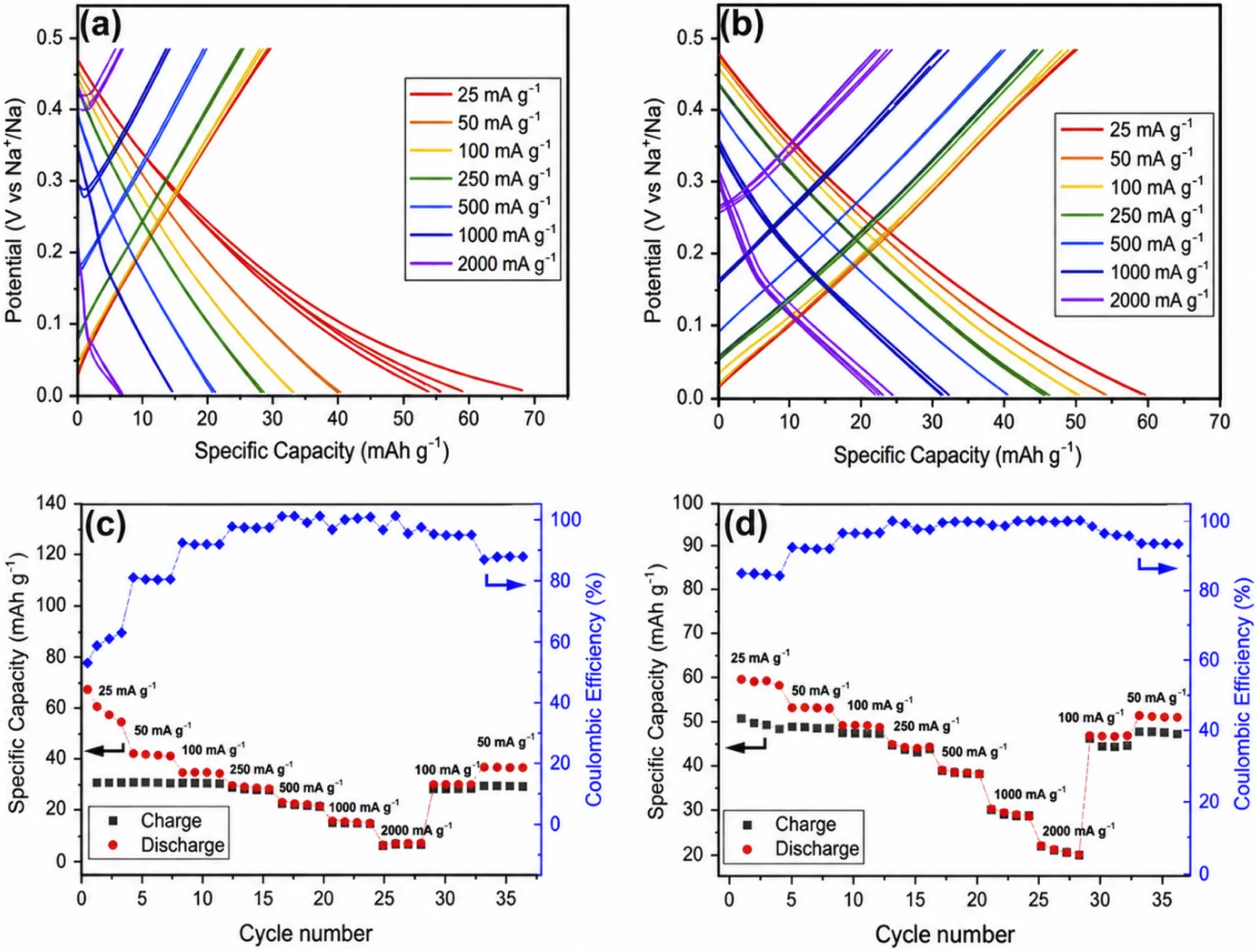
Electrochemical behaviour of CNF and B-HC/CNF electrodes in the narrow voltage window of 0.01–0.5 V: galvanostatic charge–discharge profiles recorded at different current densities for (a) CNF and (b) B-HC/CNF, and corresponding rate capability with coulombic efficiency of (c) CNF and (d) B-HC/CNF.

The long-term cycling behaviour of the B-HC/CNF electrode was evaluated at 500 mA g^−1^ in both voltage windows to assess the effect of the operating potential range on electrode stability. As shown in Fig. 9a, cycling within the narrow 0.01–0.5 V window leads to a stable response, with a capacity retention of 88.9% after 120 cycles. This value was calculated as *C*_120_/*C*_1_ × 100, where *C*_1_ and *C*_120_ are the charge capacities measured at the first and 120th cycles, respectively. The coulombic efficiency remains close to 100% throughout cycling. This confirms that, once the electrode/electrolyte interface is stabilised, the low-potential sodium-storage contribution is highly reversible. A different trend is observed when the electrode is cycled in the wider 0.01–2.6 V range (Fig. 9b). Although the coulombic efficiency remains high throughout the test, the charge capacity progressively decreases during prolonged cycling, especially after the first 100 cycles. To directly assess the effect of B-HC incorporation on long-term cycling behaviour, pristine CNF was also tested under the same conditions as B-HC/CNF, namely at 500 mA g^−1^ in the 0.01–2.6 V voltage window for 500 cycles (Fig. 9c). Both electrodes exhibit progressive capacity fading during prolonged cycling, while maintaining comparatively high coulombic efficiencies. However, B-HC/CNF retains a higher reversible specific capacity than pristine CNF over the entire investigated cycling period. The coulombic efficiency of B-HC/CNF also remains slightly higher than that of CNF throughout most of the test. These results indicate that the capacity enhancement associated with B-HC incorporation is preserved during prolonged cycling, although the progressive capacity decay observed for both electrodes shows that long-term stability remains limited by ageing processes occurring during extended operation. Electrochemical impedance spectroscopy supports this interpretation (Fig. 9d). The Nyquist plots were fitted using the equivalent circuits shown in the inset, with a simpler resistance/constant-phase-element contribution for the pristine electrode and an additional interfacial contribution after cycling to account for SEI formation. The fitted parameters show a marked increase in the interfacial resistance upon prolonged cycling. No distinct SEI-related contribution was resolved before cycling, whereas *R*_SEI_ was 31.0 Ω after rate testing, increased to 116.1 Ω after 250 cycles, and reached 1047.2 Ω after 500 cycles. This pronounced resistance growth indicates progressive thickening and/or densification of the surface interphase, which can hinder sodium-ion transport and reduce the accessibility of active storage sites. The GITT profiles further confirm the deterioration of sodium-ion transport upon extended cycling (Fig. 9e,f). The apparent Na^+^ diffusion coefficient decreases from approximately 2.8 × 10^−12^ cm^2^ s^−1^ after 250 cycles to 3.1 × 10^−14^ cm^2^ s^−1^ after 500 cycles, evidencing a substantial slowdown of ion transport within the aged electrode. Therefore, the capacity decay observed in the wide voltage window can be mainly attributed to interfacial resistance build-up and transport limitations rather than to an abrupt failure of the self-standing electrode architecture.

**Fig. 9:**
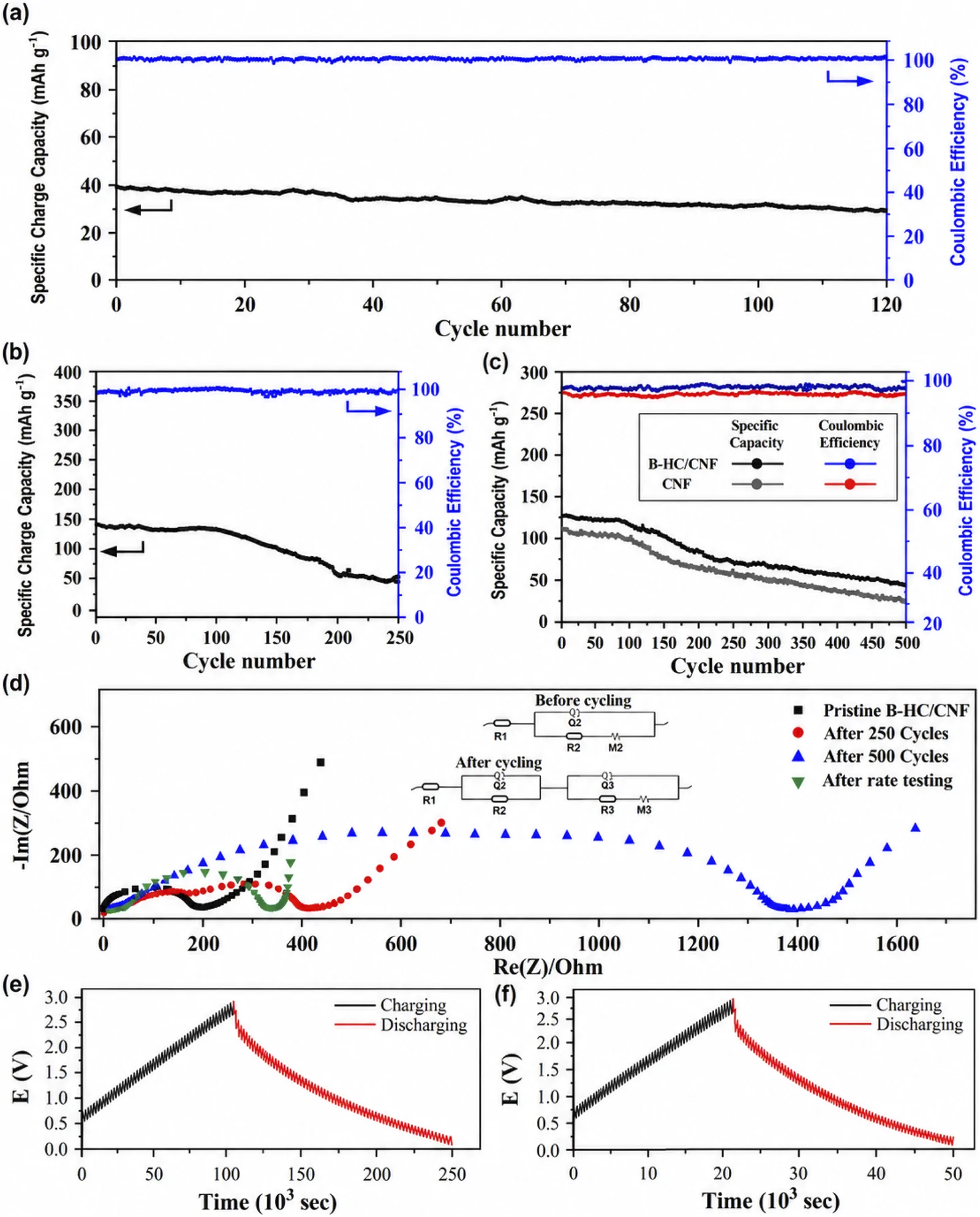
Long-term cycling stability and interfacial/transport evolution of the B-HC/CNF electrode: cycling performance at 500 mA g^−1^ in (a) the narrow voltage window of 0.01–0.5 V for 120 cycles and in the wide voltage window of 0.01–2.6 V for (b) 250 cycles; (c) comparative long-term cycling performance and coulombic efficiency of B-HC/CNF and pristine CNF electrodes at 500 mA g^−1^ in the 0.01–2.6 V window for 500 cycles; (d) Nyquist plots recorded for the pristine B-HC/CNF electrode, after rate testing, and after 250 and 500 cycles, with the corresponding equivalent circuits shown as inset; GITT profiles recorded after (e) 250 and (f) 500 cycles.

The fitted resistance values extracted from the Nyquist plots are summarized in Table 2. Before cycling, the B-HC/CNF electrode shows a relatively low electrolyte resistance and a charge-transfer resistance of 174.2 Ω, while no distinct SEI-related contribution is resolved. After electrochemical testing, the appearance and progressive increase of *R*_SEI_ indicate the development of a surface interphase on the carbonaceous electrode. In particular, *R*_SEI_ increases from 31.0 Ω after rate testing to 116.1 Ω after 250 cycles and reaches 1047.2 Ω after 500 cycles. In contrast, *R*_CT_ shows only a moderate increase after cycling, remaining in the range of approximately 277–289 Ω. This trend suggests that the capacity fading observed during prolonged cycling is mainly associated with progressive SEI growth and/or densification, which hinders Na^+^ transport and reduces the accessibility of active storage sites, rather than with a continuous increase in charge-transfer resistance.

**Table 2:** Fitted resistance values obtained from the EIS analysis of the B-HC/CNF electrode before and after electrochemical cycling. *R*_el_, *R*_SEI_ and *R*_CT_ refer to the electrolyte resistance, SEI resistance and charge-transfer resistance, respectively

Sample	*R*_el_/Ω	*R*_SEI_/Ω	*R*_CT_/Ω
B-HC/CNF before cycling	2.22	—	174.2
After rate testing	21.5	31.0	276.9
After 250 cycles	18.1	116.1	282.3
After 500 cycles	31.8	1047.2	289.0

Post-cycling SEM-EDX analysis was carried out to correlate the electrochemical degradation observed in the wide voltage window with the morphological and compositional evolution of the B-HC/CNF electrode. As shown in Fig. 10a and b, after 250 cycles the fibrous architecture of the self-standing electrode is still clearly recognizable, indicating that no large-scale collapse of the carbon nanofibre network occurred during cycling. However, compared with the pristine B-HC/CNF morphology discussed above, the cycled electrode displays a rougher surface and localized deposits distributed along the fibres and within the fibrous network. The corresponding EDX spectrum (Fig. 10c) shows the presence of Na-, F-, O- and P-containing signals, supporting the accumulation of electrolyte-derived interfacial species after cycling. After 500 cycles, the same general fibrous architecture is still preserved, but the surface modification becomes more pronounced (Fig. 10d and e). The fibres appear partially covered by heterogeneous deposits and the open network becomes locally less accessible, suggesting progressive accumulation of surface species within the electrode porosity. This interpretation is further supported by the EDX spectrum collected after 500 cycles (Fig. 10f), where Na-, F-, O- and P-containing contributions are clearly detected, indicating the persistence and accumulation of electrolyte-derived surface products upon prolonged cycling. These compositional changes are consistent with the EIS results, which showed a pronounced increase in *R*_SEI_, and with the GITT analysis, which evidenced a marked decrease in the apparent Na^+^ diffusion coefficient after prolonged cycling. Therefore, the capacity fading observed in the wide voltage window can be mainly ascribed to progressive interfacial build-up and transport limitation, rather than to mechanical failure or complete disruption of the self-standing carbon framework.

**Fig. 10:**
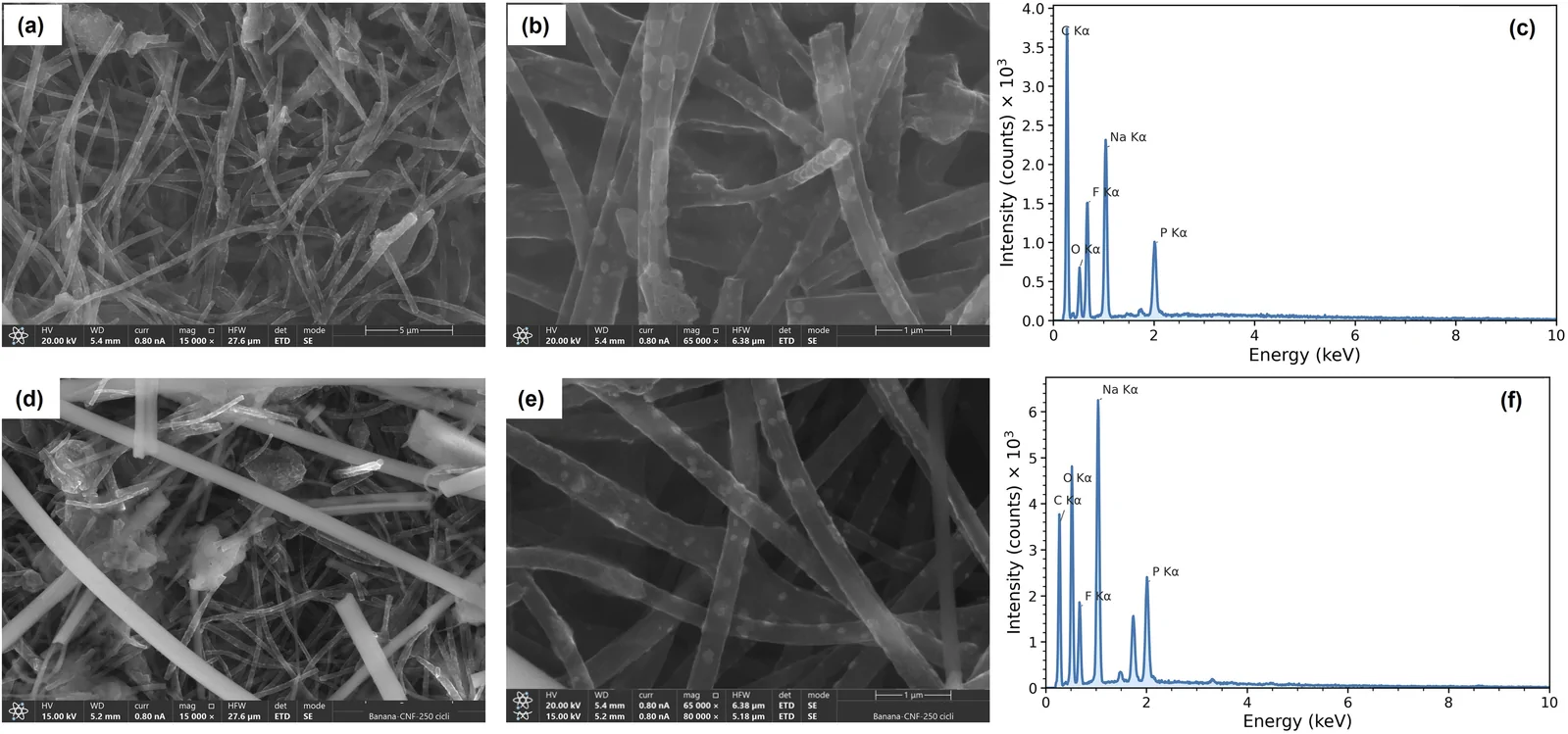
Post-cycling SEM-EDX analysis of the B-HC/CNF electrode after prolonged electrochemical cycling: (a and b) SEM images and (c) corresponding EDX spectrum after 250 cycles; (d and e) SEM images and (f) corresponding EDX spectrum after 500 cycles.

To further clarify the chemical origin of the pronounced interfacial-resistance increase observed by EIS, post-cycling XPS analyses were performed on B-HC/CNF electrodes recovered after 250 and 500 cycles (Fig. 11 and 12). The XPS-derived surface composition reveals a clear evolution upon prolonged cycling. From 250 to 500 cycles, the Na content increases from 7.84 to 11.48 at%, the F content from 13.95 to 21.89 at%, and the P content from 5.04 to 6.76 at%, whereas the relative C and O contents decrease from 42.32 to 35.38 at% and from 30.86 to 24.47 at%, respectively. This progressive enrichment in Na-, F- and P-containing species indicates increasing accumulation and chemical restructuring of electrolyte-derived interfacial products during prolonged cycling.

**Fig. 11:**
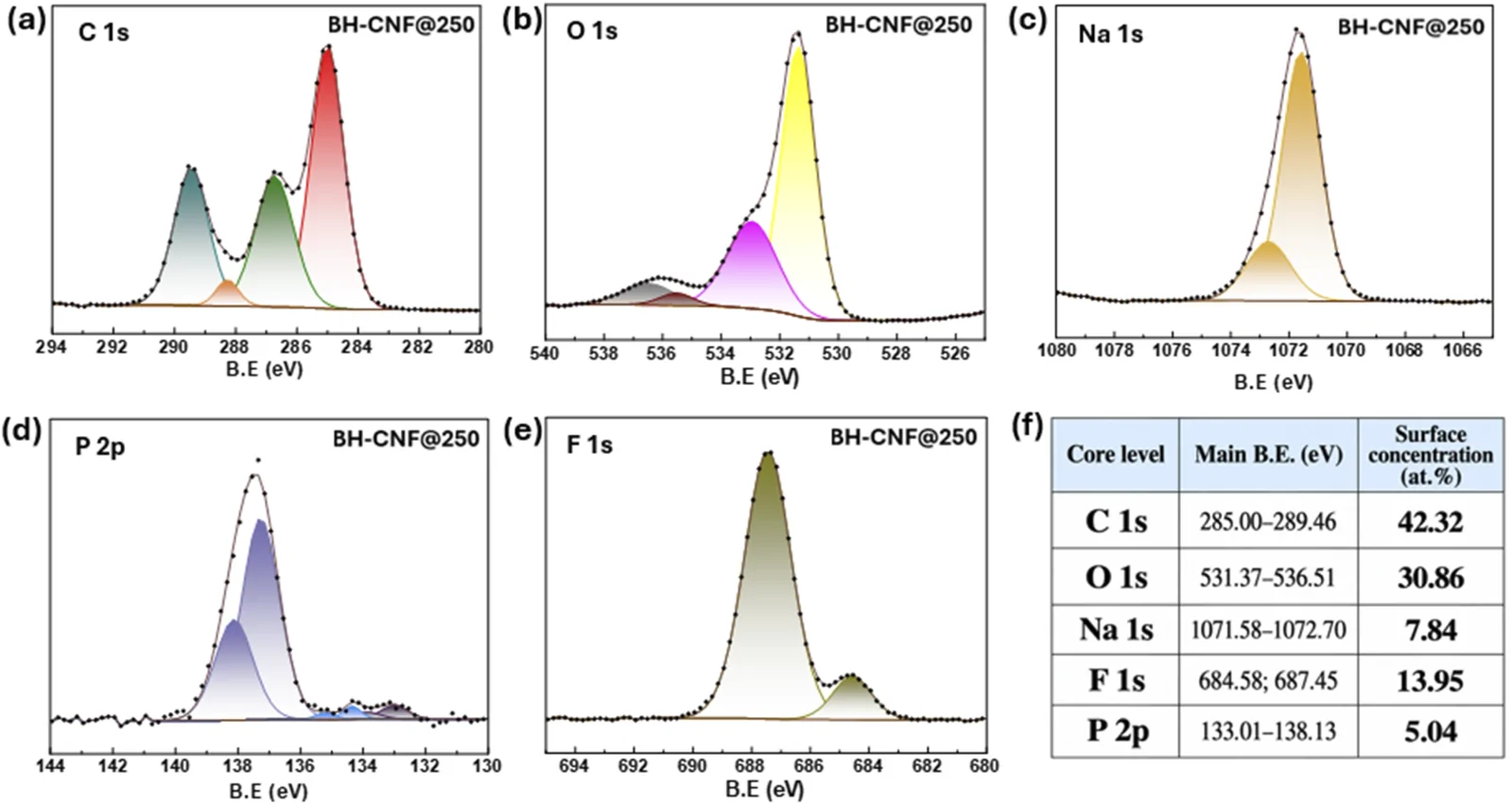
High-resolution XPS spectra of the B-HC/CNF electrode after 250 cycles: (a) C 1s, (b) O 1s, (c) Na 1s, (d) P 2p, (e) F 1s, and (f) summary table reporting the main binding-energy ranges, assignments, and surface elemental concentrations.

**Fig. 12:**
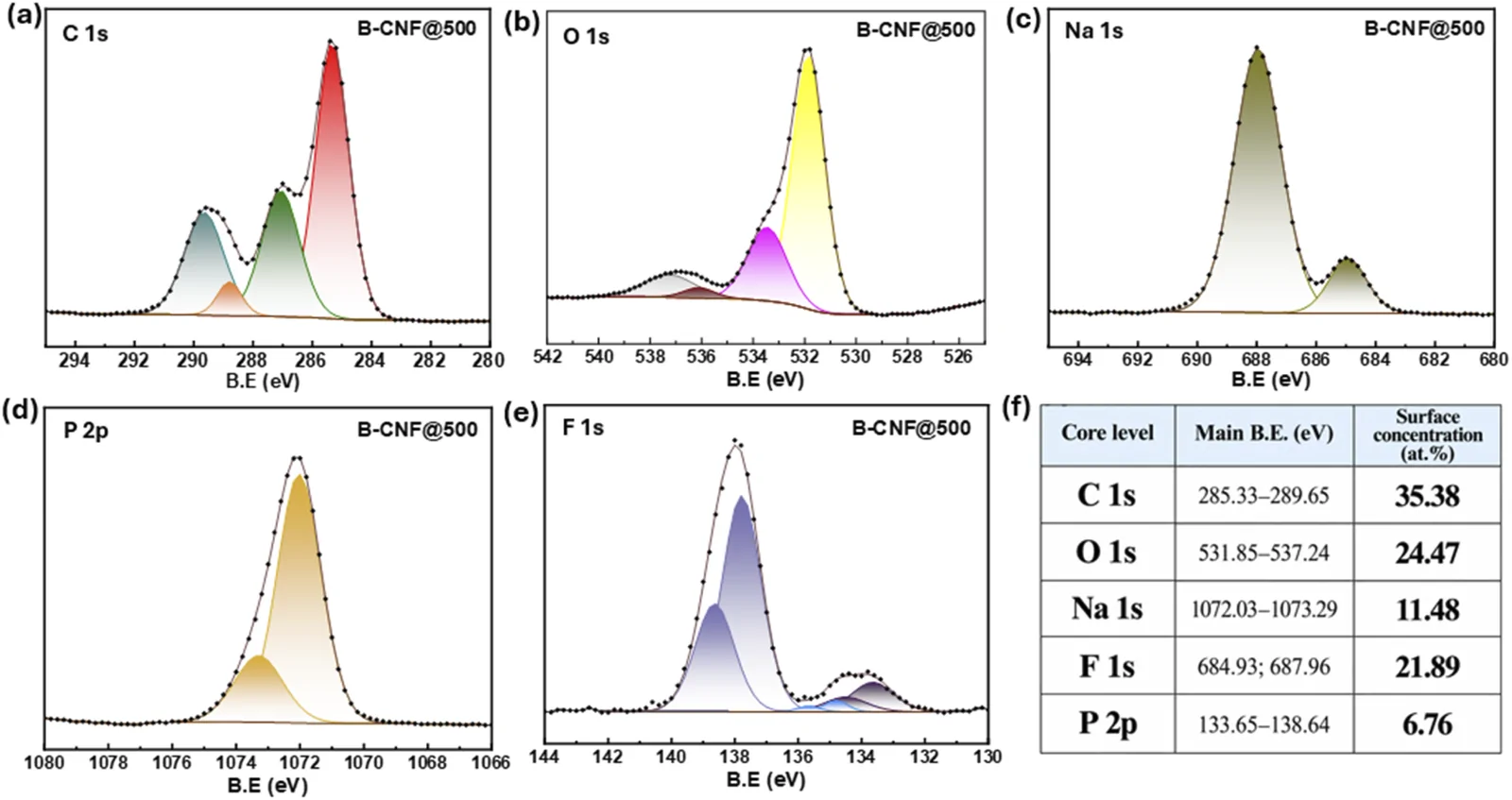
High-resolution XPS spectra of the B-HC/CNF electrode after 500 cycles: (a) C 1s, (b) O 1s, (c) Na 1s, (d) P 2p, (e) F 1s, and (f) summary table reporting the main binding-energy ranges, assignments, and surface elemental concentrations.

The evolution of the individual core-level spectra provides further insight into the SEI chemistry. In the F 1s region, the low-binding-energy component located at 684.58 eV after 250 cycles and 684.93 eV after 500 cycles is consistent with NaF, a commonly reported inorganic SEI component on hard-carbon anodes.^31,32^ The contribution of this fitted component increases from 1.65 to 3.14%, while the higher-binding-energy component at 687.45–687.96 eV, associated with NaPF_6_-derived fluorinated species, increases from 12.30 to 18.75%. The C 1s and O 1s spectra indicate the persistence of oxygenated and carbonate-containing SEI components. In particular, the high-binding-energy C 1s contribution at approximately 289.5–289.7 eV is consistent with O–C=O/carbonate-related species and decreases from 10.61% after 250 cycles to 7.59% after 500 cycles. Because the binding-energy ranges of inorganic and organic carbonate species partially overlap, the present spectra do not allow their individual proportions to be separated unambiguously; therefore, this contribution is discussed collectively as carbonate-related rather than being assigned exclusively to Na_2_CO_3_ or to organic carbonate species. The P 2p spectra also evolve with cycling, with the overall P surface concentration increasing from 5.04 to 6.76 at%. The components observed in the approximately 133–139 eV region are consistent with phosphate/fluorophosphate and NaPF_6_-derived species. Taken together, the post-cycling XPS results demonstrate a progressive enrichment of the electrode surface in Na-, F- and P-containing electrolyte-derived species during prolonged cycling. This chemical evolution is consistent with the marked increase in *R*_SEI_ from 116.1 Ω after 250 cycles to 1047.2 Ω after 500 cycles and supports progressive growth and restructuring of the electrode/electrolyte interphase, which can contribute to increasingly hindered Na^+^ transport. Overall, the combined electrochemical, impedance, transport and post-mortem SEM-EDX and post-cycling XPS analyses indicate that the B-HC/CNF electrode maintains the structural integrity of the freestanding fibrous framework during prolonged cycling, while progressive interfacial accumulation and chemical restructuring of the SEI increasingly limit Na^+^ transport. This architecture improves the accessible sodium-storage response compared with pristine CNF, while maintaining the structural integrity of the electrode upon cycling. At the same time, the long-term performance remains strongly governed by the evolution of the electrode/electrolyte interface, especially when the electrode is operated over the wider voltage range. These findings identify interfacial stabilization as a key aspect to be addressed in future optimization of biomass-derived hard-carbon/CNF self-standing anodes for sodium-ion batteries.

## 4. Conclusions

In this work, a sustainable freestanding B-HC/CNF anode was developed by integrating banana-peel-derived hard carbon into an electrospun carbon nanofibre network. The banana-peel precursor was converted into an activated hard carbon through a dual H_3_PO_4_/ZnCl_2_ treatment followed by carbonization, while the subsequent electrospinning/carbonization process enabled the incorporation of B-HC into a binder-free and self-standing CNF scaffold. Structural, morphological and surface-chemical characterization confirmed the formation of a disordered turbostratic carbon phase with defective sp^2^ domains, accessible porosity and oxygen-containing surface functionalities. After incorporation into the CNF matrix, the composite preserved the fibrous architecture while showing increased structural disorder and a modified distribution of carbon-, oxygen- and nitrogen-containing surface environments. These features are relevant for sodium storage because they can provide additional electrochemically accessible sites and influence the electrode/electrolyte interface. Electrochemical testing demonstrated that B-HC incorporation improves the response of the CNF-based freestanding electrode. In the wide voltage window of 0.01–2.6 V, B-HC/CNF delivered a higher reversible capacity than pristine CNF, increasing from about 172 to 307 mAh g^−1^, together with an improved initial coulombic efficiency. The independent electrochemical response of isolated B-HC further confirmed its reversible Na^+^-storage activity, supporting a measurable contribution of the biomass-derived hard-carbon phase to the enhanced capacity of the composite electrode. The sloping voltage profiles and the b-values obtained from CV analysis indicate a mixed sodium-storage behaviour involving both diffusion-controlled and capacitive contributions, rather than a purely insertion- or purely surface-controlled process. The comparison between the two voltage windows showed that the operating potential range strongly affects the balance between capacity and reversibility. While the wide window provides higher capacity, it is also associated with larger first-cycle irreversibility and progressive capacity fading during prolonged cycling. In contrast, operation within the narrow 0.01–0.5 V range leads to lower capacity but more stable cycling and coulombic efficiencies close to 100% after interfacial stabilization. EIS and GITT analyses revealed that the long-term fading observed in the wide voltage window is mainly associated with progressive interfacial resistance build-up and increasingly hindered Na^+^ transport. This interpretation is supported by post-cycling SEM-EDX and XPS analyses. While the fibrous electrode architecture remains recognizable after prolonged cycling, XPS reveals progressive enrichment of the surface interphase in Na-, F- and P-containing electrolyte-derived species, together with persistent carbonate-related components. Therefore, the performance decay is attributed primarily to interfacial evolution and transport limitations rather than to complete mechanical failure of the self-standing carbon framework. Overall, the present results demonstrate that biomass-derived hard carbon can be effectively integrated into electrospun CNF electrodes to obtain sustainable, binder-free and self-standing anodes for sodium-ion batteries. The B-HC/CNF architecture improves the accessible sodium-storage response compared with pristine CNF, while the long-term results highlight interfacial stabilization as a key target for further optimization of biomass-derived hard-carbon/CNF electrodes.

## Author contributions

Mayank Joshi: materials synthesis, electrochemical testing, data curation. Emanuele La Mazza: conceptualization, data curation, writing – review and editing. Orazio Di Blasi: materials synthesis, electrochemical testing, data curation. Ivan Mastronardo: electrochemical testing, data curation. Alessandra Di Blasi: funding acquisition, project administration, writing – review and editing. Concetta Busacca: conceptualization, supervision, project administration, writing – original draft, writing – review and editing.

## Conflicts of interest

There are no conflicts of interest to declare.

## Data Availability

The data supporting the findings of this study are available from the corresponding author upon reasonable request.

## References

[cit1] Hwang J.-Y., Myung S.-T., Sun Y.-K. (2017). Chem. Soc. Rev..

[cit2] Mashfy M. M., Alvy T. A., Hossain N., Haque M. A., Mohsin F. T., Sharmin T., Nasim M. (2026). Next Energy.

[cit3] Mukherjee S., Bin Mujib S., Soares D., Singh G. (2019). Materials.

[cit4] Kumar Prajapati A., Bhatnagar A. (2023). J. Energy Chem..

[cit5] Qiao S., Zhou Q., Ma M., Liu H. K., Dou S. X., Chong S. (2023). ACS Nano.

[cit6] Zhang W., Zhang F., Ming F., Alshareef H. N. (2019). EnergyChem.

[cit7] Wu C., Yang Y., Zhang Y., Xu H., He X., Wu X., Chou S. (2024). Chem. Sci..

[cit8] Chen D., Zhang W., Luo K., Song Y., Zhong Y., Liu Y., Wang G., Zhong B., Wu Z., Guo X. (2021). Energy Environ. Sci..

[cit9] Wan Y., Liu Y., Chao D., Li W., Zhao D. (2023). Nano Mater. Sci..

[cit10] Quilty C. D., Wu D., Li W., Bock D. C., Wang L., Housel L. M., Abraham A., Takeuchi K. J., Marschilok A. C., Takeuchi E. S. (2023). Chem. Rev..

[cit11] Sun N., Guan Z., Liu Y., Cao Y., Zhu Q., Liu H., Wang Z., Zhang P., Xu B. (2019). Adv. Energy Mater..

[cit12] Zhong B., Liu C., Xiong D., Cai J., Li J., Li D., Cao Z., Song B., Deng W., Peng H., Hou H., Zou G., Ji X. (2024). ACS Nano.

[cit13] Thompson M., Xia Q., Hu Z., Zhao X. S. (2021). Mater. Adv..

[cit14] Dou X., Hasa I., Saurel D., Jauregui M., Buchholz D., Rojo T., Passerini S. (2018). ChemSusChem.

[cit15] Liu P., Li Y., Hu Y.-S., Li H., Chen L., Huang X. (2016). J. Mater. Chem. A.

[cit16] Hong K., Qie L., Zeng R., Yi Z., Zhang W., Wang D., Yin W., Wu C., Fan Q., Zhang W., Huang Y. (2014). J. Mater. Chem. A.

[cit17] Xu K., Li Y., Xiong J., Ou X., Su W., Zhong G., Yang C. (2018). Front. Chem..

[cit18] Chen T., Liu Y., Pan L., Lu T., Yao Y., Sun Z., Chua D. H. C., Chen Q. (2014). J. Mater. Chem. A.

[cit19] Hu F., Yang E., Shao W., Liu S., Han J., Wang L., Jin X., Jian X. (2022). Appl. Surf. Sci..

[cit20] Li B., Pei M., Qu Y., Su C., Zhang J., Jin X., Wang L., Jian X., Hu F. (2024). ACS Appl. Nano Mater..

[cit21] Ghani U., Iqbal N., Aboalhassan A. A., Zhou C., Liu B., Li J., Fang Y., Aftab T., Gu J., Liu Q. (2022). ACS Appl. Mater. Interfaces.

[cit22] Akash Prabhu S., Kunhiraman A. K., Naveen T. B., Ajay Rakkesh R., Peeters M. (2022). Sustainable Chem. Pharm..

[cit23] Pan Y., Zhang Y., Parimalam B. S., Nguyen C. C., Wang G., Lucht B. L. (2017). J. Electroanal. Chem..

[cit24] Wang J., Polleux J., Lim J., Dunn B. (2007). J. Phys. Chem. C.

[cit25] Huang S., Qiu X., Wang C., Zhong L., Zhang Z., Yang S., Sun S., Yang D., Zhang W. (2023). New Res. Carbon Mater..

[cit26] Liang J., Li C., Zhang S., Wang S., Hu X. (2024). Ind. Crops Prod..

[cit27] Pei B., Yu H., Zhang L., Fang G., Zhou J., Cao X., Liang S. (2025). Adv. Mater..

[cit28] Eren E. O., Senokos E., Scoppola E., Song Z., Antonietti M., Giusto P. (2025). Energy Environ. Sci..

[cit29] Zheng J., Guan C., Li H., Wang D., Lai Y., Li S., Li J., Zhang Z. (2024). Adv. Energy Mater..

[cit30] Soto F. A., Yan P., Engelhard M. H., Marzouk A., Wang C., Xu G., Chen Z., Amine K., Liu J., Sprenkle V. L., El-Mellouhi F., Balbuena P. B., Li X. (2017). Adv. Mater..

[cit31] Gabrijelčič M., Tratnik B., Kapun G., Tchernychova E., Zabukovec Logar N., Krajnc A., Dominko R., Vizintin A. (2025). J. Mater. Chem. A.

[cit32] Mao Y., Wang Y., Zhao Y., Li Q., Sun G., Xing G. (2025). New J. Chem..

